# 2D-Self-Assembled Organic Materials in Undoped Hole Transport Bilayers for Efficient Inverted Perovskite Solar Cells

**DOI:** 10.1021/acsami.2c23010

**Published:** 2023-04-26

**Authors:** Isaac G. Sonsona, Manuel Carrera, Miriam Más-Montoya, Rafael S. Sánchez, Patricio Serafini, Eva M. Barea, Iván Mora-Seró, David Curiel

**Affiliations:** † Department of Organic Chemistry, Faculty of Chemistry, University of Murcia, 30100 Murcia, Spain; ‡ Institute of Advanced Materials, University Jaume I, Avenida de Vicent Sos Baynat, s/n, 12071 Castelló de la Plana, Spain

**Keywords:** self-assembly, interface engineering, hole transporting material, perovskite solar cell

## Abstract

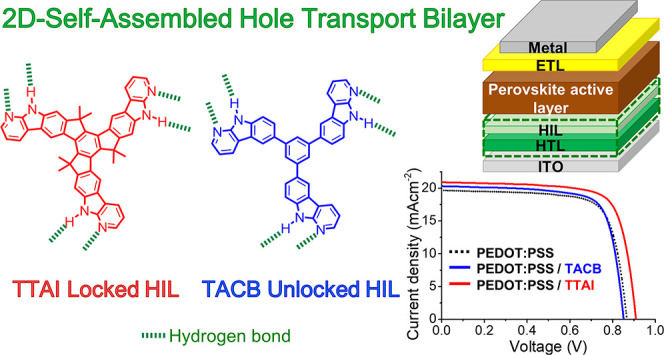

Interfaces between photoactive perovskite layer and selective contacts play a key role in the performance of perovskite solar cells (PSCs). The properties of the interface can be modified by the introduction of molecular interlayers between the halide perovskite and the transporting layers. Herein, two novel structurally related molecules, 1,3,5-tris(α-carbolin-6-yl)benzene (**TACB**) and the hexamethylated derivative of truxenotris(7-azaindole) (**TTAI**), are reported. Both molecules have the ability to self-assemble through reciprocal hydrogen bond interactions, but they have different degrees of conformational freedom. The benefits of combining these tripodal 2D-self-assembled small molecular materials with well-known hole transporting layers (HTLs), such as PEDOT:PSS and PTAA, in PSCs with inverted configuration are described. The use of these molecules, particularly the more rigid **TTAI**, enhanced the charge extraction efficiency and reduced the charge recombination. Consequently, an improved photovoltaic performance was achieved in comparison to the devices fabricated with the standard HTLs.

## Introduction

The first photovoltaic device based on an organic–inorganic hybrid perovskite material was reported by Miyasaka and colleagues in 2009.^1^ The power conversion efficiency (PCE) of that pioneer solar cell was merely of 3.8%. Since then, the efficiency of perovskite solar cells (PSCs) has remarkably improved to reach a certified record of 25.7%.^2^ The extraordinary properties of perovskite (wide sunlight absorption range with high extinction coefficients,^3^ low exciton binding energy,^4^ ambipolar charge transport with high mobilities,^5,6^ long charge diffusion length,^7^ low trap density,^8^ and versatile processability)^9^ have made these materials a promising alternative for the future generation of solar cells. More importantly, these properties admit the possibility of being further tuned and optimized through the perovskite composition.^10^ Beyond halide perovskite intrinsic properties, the role of interfaces has been crucial for the development of high performance PSCs.^11^ In this sense, the device engineering has also allowed the continuous improvement of the performance of PSCs,^12,13^ where it has been shown to be the critical effect of the interfaces between photoactive perovskite layer and transport layers. Consequently, it is worth highlighting the contribution of interfacial layers, deposited between the active perovskite film and the transport layers, hereafter denoted as hole interfacial layer (HIL), to the progress of PSCs.^14,15^ These layers play critical roles for the optimum performance of the solar cell such as control of the selective charge carrier transport, protection of the active layer, and modulation of the perovskite morphology. To accomplish these functions, interfacial layers must meet certain requirements: transparency to the solar radiation; adequate and balanced charge transport; good energy level alignment with adjacent materials; soft and homogeneous thin-film forming ability compatible with the perovskite layer processing. A myriad of organic (small molecules and polymers) and inorganic materials have demonstrated their suitability to improve the solar cell performance.^16−21^ In general, the best results have been reported for devices incorporating doped interfacial layers, particularly in the case of hole transporting materials.^22^ However, it is known that dopants commonly affect the degradation of the PSCs,^23−25^ and, accordingly, the research focused on dopant-free HTLs has taken much interest.^26−30^

Among the different PSCs configurations, the inverted device architecture (p-i-n) shows, in general, lower efficiencies, with certified PCE record of 23.6%,^31^ than the conventional configuration (n-i-p). Nevertheless, the former possesses the great advantage of requiring lower annealing temperatures during the fabrication, which enables the manufacturing of flexible devices that are compatible with roll-to-roll processing for mass production.^32^ Interestingly, these convenient processing features widen the assortment of adequate materials and procedures for the sequential deposition of multilayers. Moreover, these methodologies are suitable for the fabrication of tandem devices.^33^ Currently, the efficiency of inverted solar cells still lags behind that of the conventional devices, mainly due to lower *V*_OC_ and nonradiative recombination losses. Since these features are related to interfacial defects, the engineering of interfacial layers can efficiently contribute to the optimization of inverted PSCs. In this regard, we have focused our attention in the two most frequently used hole transporting materials in inverted PSCs, namely poly(3,4-ethylenedioxythiophene):poly(styrenesulfonate) (PEDOT:PSS)^34^ and poly[bis(4-phenyl)(2,4,6-trimethylphenyl)amine] (PTAA).^35^ Despite the common use of these materials, the hygroscopic and acidic nature of PEDOT:PSS affects the stability of the PSC. Concerning PTAA, it presents issues related to the surface energy and wettability required for the subsequent deposition of the perovskite layer, thus affecting the fabrication and corresponding performance of the PSCs. Accordingly, the deposition of an interfacial layer between the HTL and the perovskite can contribute to minimize these problems and allow a fine-tuning at the interface toward an optimum energy level matching between adjacent materials. This has been demonstrated for PEDOT:PSS and/or PTAA in hole transporting bilayers (HTBLs),^36,37^ where the detrimental charge recombination has been notably reduced by inserting inorganic materials such as copper salts (CuSCN or CuI),^38,39^ metal oxides (V_2_O_5_, NiO_X_, or MoO_3_), or phosphorene.^40^ The combination with polyelectrolytes or functionalized reduced graphene oxide has also led to improved performances.^41−43^ Nevertheless, with a few exceptions,^44,45^ organic small molecules have been barely explored for this purpose.

**Scheme 1:**
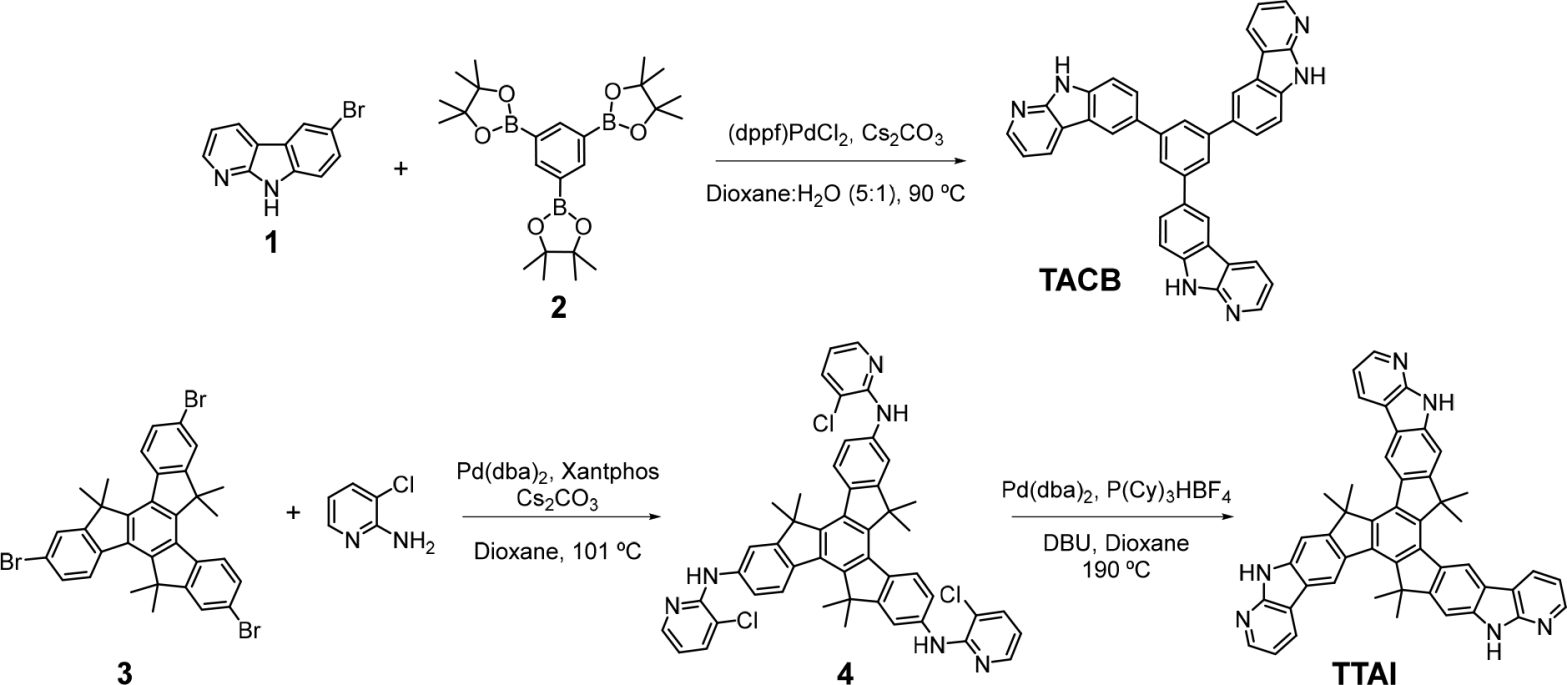
Synthetic Route for **TACB** and **TTAI**

In this work, we present a comparative study between two tripodal small molecules that can be easily integrated in the inverted PSC configuration. The use of these interfacial layers improves the performance of inverted PSCs using both PEDOT:PSS and PTAA as HTLs. We have previously demonstrated that hydrogen bond-directed self-assembly of conjugated systems represent a useful approach to increase the robustness of organic semiconductors and to preserve the structural integrity of these materials.^46,47^ Moreover, we have also proved how self-assembly can be used to control the molecular arrangement in the solid state thanks to the strength of hydrogen bonding between rationally located donor and acceptor sites and how self-assembled HTLs benefit the performance of PSCs.^48,49^ This approach has been further adapted to the development of 2D materials that can extend the self-organization of the molecules within planar domains.^50^ Based on these results, herein we have investigated the use of 2D-self-assembled π-expanded tripodal molecules as dopant-free HILs. Their condition of small molecules enables deposition by thermal evaporation that improves the control of the thickness and morphology of the thin film. Additionally, their ability to self-assemble remarkably decreases the solubility of these molecules, favoring their compatibility with the solution processing of subsequent top-layers in the solar cell. With the aim of going a step forward in the understanding of the structure–property relationship in self-assembled materials, we have synthesized two analogous molecules, namely 1,3,5-tris(α-carbolin-6-yl)benzene (**TACB**) and the 5,5,10,10,15,15-hexamethylated derivative of truxenotris(7-azaindole) (**TTAI**) (Scheme 1), that integrate three 7-azaindole units for directing the 2D-self-assembly through reciprocal hydrogen bonds but differ in the rotational freedom of the three arms. Since the planarity and the conformational freedom are important structural features when dealing with the charge transport properties of conjugated molecular systems,^51−53^ we investigate this effect on hole transporting bilayers. To this end, the rotationally free α-carboline fragments, connected to a central benzene core in **TACB**, are locked in **TTAI** by linking the central benzene and each peripheral substructure with three carbon bridges, leading to a truxene-like core. As will be discussed further, both materials exhibited suitable properties to be incorporated as interfacial layers in PSCs. Moreover, we have observed that the use of these self-assembled 2D materials in HTBLs improves the charge extraction efficiency and reduces the charge recombination, resulting in a better performance of inverted perovskite solar cells when compared to the devices fabricated with the standard HTLs.

## Experimental Section

### General

Reagents and solvents used for the synthesis, purification, and characterization of the reported compounds were commercially available and used without any purification treatment. Unless stated otherwise, all reactions were performed under nitrogen atmosphere. Final products were purified by gradient sublimation (pressure <10^–6^ mbar) to obtain high purity materials. ^1^H NMR and ^13^C NMR spectra were recorded at room temperature on a Bruker AV400 or AV300 spectrometer having frequencies of 400 or 300 MHz for proton nuclei and 101 or 75.5 MHz for carbon nuclei. The residual peak of the deuterated solvent was used as reference for the chemical shifts. Mass spectra were measured on an HPLC-MS TOF 6220 instrument. Melting points were measured in a Reichert instrument and are not corrected. Thermogravimetric analysis (TA) was performed on a SDT 2960 analyzer from TA Instruments under inert atmosphere (heating rate: 10 °C min^–1^). Differential scanning calorimetry (DSC) was performed on a TA Instrument DSC 2920, and the second heating and cooling cycle was analyzed. Absorption spectra were measured on a Cary 5000 UV–vis-NIR spectrophotometer. Emission spectra were recorded on a Jobin Yvon Fluorolog 3–22 luminescence spectrometer using a 450-W xenon lamp, double grating monochromators, and a TBX-04 photomultiplier. Cyclic voltammetry experiments were performed in dichloromethane using a BAS potentiostat. A platinum coated electrode was employed as working electrode, a Pt wire as counter electrode, Ag/AgCl as reference electrode, and the ferrocene/ferrocenium (Fc/Fc^+^) couple as internal reference. The scan rate was 100 mV s^–1^. Tetrabutylammonium hexafluorophosphate (0.1 M) was the supporting electrolyte.

### Synthetic Methods

#### 1,3,5-Tris(α-carbolin-6-yl)benzene, **TACB**

6-Bromo-α-carboline (494 mg, 2 mmol), 1,3,5-tris(4,4,5,5-tetramethyl-1,3,2-dioxaborolan-2-yl)benzene (273 mg, 0.6 mmol), dichloro[1,1′-bis(diphenylphosphino)ferrocene]palladium(II) (76.6 mg, 0.1 mmol), and cesium carbonate (795 mg, 2.4 mmol) were introduced in a Schlenk flask, and nitrogen atmosphere was made to the system through vacuum/nitrogen cycles. In another flask, a mixture of water (10 mL) and 1,4-dioxane (50 mL) was bubbled with nitrogen for 15–20 min. Then this mixture was added to the Schlenk flask and nitrogen bubbling was continued for 10 additional minutes. Finally, the reaction mixture was introduced in preheated oil bath at 90 °C and it was allowed to react at that temperature with magnetic stirring. After 60 h, the Schlenk flask was cooled down to room temperature and water was added to the reaction mixture, obtaining a suspension that was stirred for 10 min. Then the solid was collected by filtration, washed with water, and dried. The brown solid obtained was purified by silica gel column chromatography using THF as eluent, followed by trituration with MeOH and CH_2_Cl_2_, yielding the desired product as a light yellow solid (127 mg, 37% yield). Mp > 300 °C. ^1^H NMR (400 MHz, DMSO-*d*_*6*_), δ: 11.90 (s, 3H), 8.79 (d, *J* = 1.5 Hz, 3H), 8.67 (dd, *J* = 7.7, 1.5 Hz, 3H), 8.45 (dd, *J* = 4.8, 1.6 Hz, 3H), 8.11 (s, 3H), 8.07 (dd, *J* = 8.5, 1,8 MHz, 3H), 7.66 (d, *J* = 8.4 Hz, 3H), 7.26 (dd, *J* = 7.7, 4.8 Hz, 3H) ppm. ^13^C NMR (APT) (75.5 MHz, DMSO-*d*_*6*_), δ: 152.4 (C), 146.3 (CH), 142.4 (C), 138.5 (C), 132.2 (C), 128.9 (CH), 126.1 (CH), 123.7 (CH), 121.2 (C), 119.9 (CH), 115.6 (C), 115.2 (CH), 111.7 (CH) ppm. HRMS (ESI), *m*/*z*, [M + H]^+^ Calcd for C_39_H_25_N_6_: 577.2135, Found: 577.2148.

#### 5,5,10,10,15,15-Hexamethyl-N^2^,N^7^,N^12^-tris(3-chloropyridin-2-yl)truxene-2,7,12-triamine, **4**

In a two-neck round-bottom flask under nitrogen atmosphere, stirring, and at room temperature, dry dioxane (20 mL) was added via syringe to a mixture of compound **3** (330 mg, 0.50 mmol), 2-amino-3-chloropyridine (384 mg, 2.99 mmol), bis(dibenzylideneacetone)palladium (0) (14.3 mg, 0.025 mmol), 4,5-bis(diphenylphosphino)9,9-dimethylxanthene (Xantphos) (21.6 mg, 0.037 mmol), and cesium carbonate (0.97 g, 2.99 mmol). The reaction was heated at reflux temperature and the progress of the reaction monitored by thin layer chromatography. After 24 h, Pd(dba)_2_ (14.3 mg, 0.025 mmol) and Xantphos (21.6 mg, 0.037 mmol) were added again and the reaction was allowed to continue for 24 more hours. After this time, the reaction flask was cooled down and the solvent removed under vacuum. The resulting crude was purified by silica gel column chromatography (Hexane/THF, 7/3) to obtain the product as a yellow solid (267 mg, 67% yield). Mp: 298 °C. ^1^H NMR (400 MHz, CDCl_3_), δ: 8.26 (d, *J* = 8.6 Hz, 3H), 8.22 (dd, *J* = 4.8, 1.6 Hz, 3H), 7.83 (dd, *J* = 8.6, 2.2 Hz, 3H), 7.69 (d, *J* = 2.1 Hz, 3H), 7.62 (dd, *J* = 7.7, 1.6 Hz, 3H), 7.21 (s, 3H), 6.76 (dd, *J* = 7.7, 4.9 Hz, 3H), 1.91 (s, 18H) ppm. ^13^C NMR (APT) (75.5 MHz, C*D*Cl_3_), δ: 158.9 (C), 151.4 (C), 146.6 (C), 146.1 (CH), 138.6 (C), 136.8 (CH), 135.6 (CH), 131.8 (C), 126.2 (CH), 118.0 (CH), 116.3 (C), 115.3 (CH), 114.0 (CH), 47.0 (C), 24.3 (CH_3_) ppm. HRMS (ESI) *m*/*z*: [M + H]^+^ Calcd for C_48_H_40_Cl_3_N_6_: 807.2357; Found: 807.2358.

#### 5,5,10,10,15,15-Hexamethyltruxeno[2,3-b:7,8-b′:12,13-b′′]tri(7-azaindole), **TTAI**

The reaction was performed in a sealed tube previously loaded with dry dioxane (8.5 mL), compound **4** (230 mg, 0.29 mmol), bis(dibenzylideneacetone)palladium (0) (98.3 mg, 0.17 mmol), tricyclohexylphosphonium tetrafluoroborate (127 mg, 0.342 mmol), and 1,8-diazabicylo(5.4.0)undec-7-ene (0.40 mL, 2.72 mmol). After the reaction mixture was bubbled with nitrogen for 10 min, the tube was sealed and the reaction was stirred for 5 days at 190 °C. Once the reaction cooled down, water (50 mL) was added to obtain a yellow suspension that was filtered under vacuum and sequentially washed with water (3 × 50 mL), diethyl ether (3 × 10 mL), and chloroform (3 × 20 mL). The pure product was isolated as a pale gray solid (156 mg, 79% yield). Mp: > 300 °C. ^1^H NMR (400 MHz, CDCl_3_ (1% TFA-*d*)), δ: 13.22 (brs, 3H), 9.07 (s, 3H), 9.04 (dd, *J* = 7.5, 0.7 Hz, 3H), 8.47 (dd, *J* = 5.9, 0.8 Hz, 3H), 8.07 (s, 3H), 7.63 (dd, *J* = 7.6, 6.1 Hz, 3H), 2.12 (s, 18H) ppm. ^13^C NMR (APT) (75.5 MHz, C*D*Cl_3_ (1% TFA-*d*)), δ: 161.3 (C), 148.1 (C), 145.6 (C), 139.4 (C), 135.6 (C), 134.9 (CH), 133.3 (CH), 132.3 (C), 123.7 (C), 118.5 (C), 118.2 (CH), 114.8 (CH), 108.1 (CH), 47.3 (C), 24.6 (CH_3_) ppm. HRMS (ESI) *m*/*z*: [M-H]^−^ Calcd for C_48_H_35_N_6_: 695.2929; Found: 695.2923.

#### X-ray Diffraction

Single crystals of **TACB** suitable for X-ray diffraction measurements were grown by slow evaporation of a DMSO solution. Intensities were registered at low temperature on a Bruker D8QUEST diffractometer using monochromated Cu Kα radiation (λ = 1.54178 Å). Absorption corrections were based on multiscans (program SADABS). Structures were refined anisotropically using SHELXL-2018. Hydrogen atoms were included using a riding model. The NH hydrogen was located in a difference synthesis and was refined as free. CCDC 2225042 contains the supplementary crystallographic data for **TACB**. These data can be obtained free of charge from The Cambridge Crystallographic Data Centre via www.ccdc.cam.ac.uk/structures.

## Results and Discussion

### Synthesis and Characterization

The synthetic routes designed for the novel small molecules are outlined in Scheme 1. The more conformationally free **TACB** was obtained via a 3-fold Suzuki-Miyaura^54^ cross-coupling reaction between 6-bromo-α-carboline (**1**)^55,56^ and the corresponding 1,3,5-tris(4,4,5,5-tetramethyl-1,3,2-dioxaborolan-2-yl)benzene, **2**. As far as the conformationally locked **TTAI** synthesis is concerned, first, the 5,5,10,10,15,15-hexamethyl-2,7,12-tribromotruxene, **3**,^57,58^ was subject to a triple Pd-catalyzed Buchwald-Hartwig^59^ C–N coupling with 2-amino-3-chloropyridine. Subsequently, a Pd-mediated intramolecular cyclization of the resulting product, **4**, afforded the rigid skeleton of **TTAI**. The structure of all the intermediate and final products was confirmed by proton and carbon nuclear magnetic resonance spectroscopies and high-resolution mass spectrometry. Further details can be found in the Supporting Information. Additionally, final products were subject to gradient sublimation to obtain highly pure materials.

**Figure 1:**
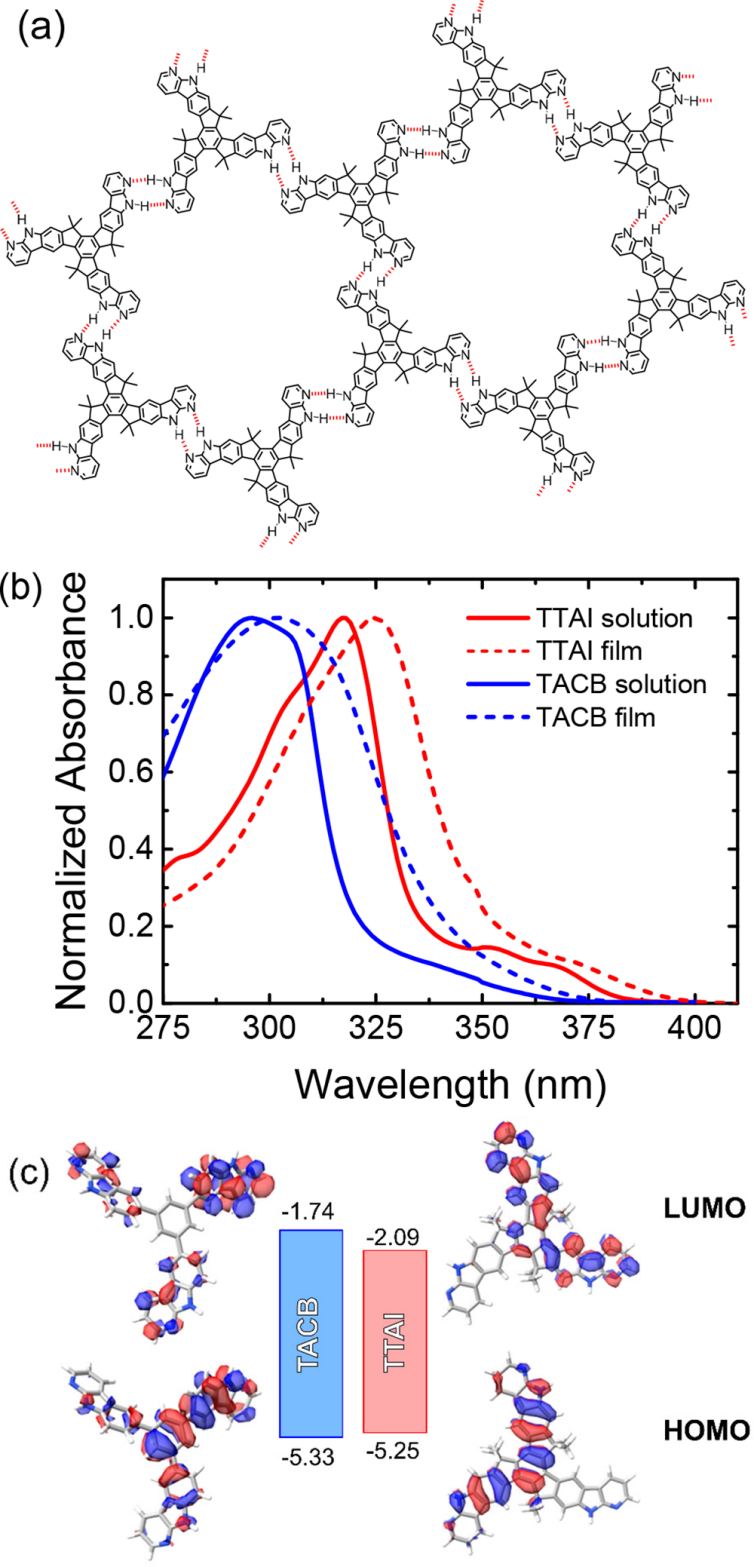
(a) Idealized view of 2D-self-assembled **TTAI**. (b) Normalized absorption spectra in DMF solution and as thin film. (c) Energy diagram and computed frontier molecular orbitals (0.02 isovalue). Experimental HOMO and LUMO energy values are given in eV.

Density functional theory (DFT) calculations confirmed the conformational differences between **TACB** and **TTAI**. The three peripheral α-carbolines in **TACB** are not coplanar but are rotated (36.8–39.5°) with respect to the plane defined by the central benzene core to minimize the steric hindrance (Figure S9a). Conversely, the fused polyheteroaromatic structure of **TTAI** presents a completely planar geometry (Figure S9b). Besides, three 7-azaindole units have been rationally located in the periphery of the conjugated systems, **TACB** and **TTAI**, to direct the 2D-supramolecular self-assembly through reciprocal hydrogen bonding between the hydrogen bond donor sites (N–H_pyrrole_) and the hydrogen bond acceptor sites (N_pyridine_) (Figure 1a). This building bock has been previously demonstrated to be an excellent synthon for controlling the molecular arrangement in the solid state.^47,49,60^ Hydrogen bond-directed self-assembly has also been confirmed by the single-crystal X-ray diffraction of **TACB** (Figure S10). The supramolecular self-assembly bestows a significant thermal robustness to the novel materials that display high decomposition temperatures of 508 °C (**TACB**) and 512 °C (**TTAI**) (Figure S7).

The optical properties of the materials were analyzed by absorption spectroscopy (Figure 1b and Table 1). **TACB** showed a broad band at 296 nm, ascribed to π–π* transitions. Concerning **TTAI**, the most prominent band is bathochromically shifted to 317 nm due to the more extended conjugation in this conformationally locked molecule. Besides, a small shift toward lower energy was detected in the thin film spectra with respect to those measured in solution owing to the stronger intermolecular interactions established in the solid state. In any case, it is worth noticing that no absorption was observed above 400 nm, making these molecules transparent to the visible radiation. This feature implies that the novel molecules are good candidates to be incorporated as interfacial materials since they will not interfere with the sunlight absorption at the perovskite layer. Consequently, wide optical gaps were estimated from the onset of the lowest energy band of the thin film absorption spectra with values of 3.59 and 3.16 eV for **TACB** and **TTAI**, respectively.

**Table 1:** Optical and Electrochemical Properties

material	λ_max._^abs. sol.^ [nm]	λ_max._^abs. film^ [nm]	*E*_g_^film^ [eV]^a^	*E*_HOMO_ [eV]^b^	*E*_LUMO_ [eV]^c^
**TACB**	296	302	3.59	–5.33	–1.74
**TTAI**	317	325	3.16	–5.25	–2.09

a *E*_g_^film^ [eV] = 1240/ λ_onset_^film^ [nm].

b *E*_HOMO_ = −4.8 – (*E*_onset_ – *E*_Fc, onset_).

c *E*_LUMO_ = *E*_HOMO_ + *E*_g_.

Cyclic voltammetry showed irreversible voltammograms for both compounds, with **TACB** presenting a higher anodic peak potential (1078 mV) than the rigid **TTAI** (1064 mV) (Figure S11). Consequently, a slightly deeper HOMO energy was estimated from the oxidation onset of **TACB** (−5.33 eV) than **TTAI** (−5.25 eV) (Figure 1b). The LUMO energies (**TACB**: – 1.74 eV; **TTAI**: – 2.09 eV) were calculated by adding the optical gaps to the HOMO energy. Additionally, DFT theoretical studies allowed the determination of the isosurfaces of the frontier molecular orbitals (Figure 1c). The spatial representation of the HOMO on the **TACB** structure revealed a larger contribution of the central benzene and of one of the α-carboline units. The LUMO isosurface also displayed larger coefficients localized in one of the arms of the tripodal structure with the other two showing a smaller contribution and without a clear participation of the benzene core. As far as **TTAI** is concerned, the HOMO is mainly distributed over two arms of the molecule and this also occurs for the LUMO isosurface.

The estimated energy levels present a correct alignment with the HOMO energies of PEDOT:PSS (−5.1 eV) and PTAA (−5.2 eV), as well as with the valence band (−5.9 eV) of the triple-cation perovskite, that will be used as active layer (Figure S12). The different combinations of HTBLs formed with each molecular material, **TACB** and **TTAI**, define an energy gradient that could improve the driving force for the hole extraction (Figure S12). Additionally, the high LUMO energies confirm the suitability of these materials to block wrongly directed electrons.

Charge transport measurements have been performed on single carrier devices fabricated with the architecture ITO/MoO_3_/HTM/MoO_3_/Ag. The *J*–*V* characteristics corresponding to the space-charge-limited current regime have been fitted to the Murgatroyd model (Figure S13).^61^ As it could be inferred from DFT models, showing a planar skeleton for **TTAI** and a twisted structure for **TACB**, the hole mobility determined for **TTAI** (4.8 × 10^–5^ cm^2^ V^–1^ s^–1^) is almost 2 orders of magnitude higher than for **TACB** (7.1 × 10^–7^ cm^2^ V^–1^ s^–1^), presumably due to the better π–π intermolecular interactions within the bulk hydrogen bonded material.

### Solar Cells Fabrication and Characterization

To explore the ability of the novel compounds to work as HIMs, they were incorporated to devices with inverted architecture according to the sequence: indium tin oxide (ITO)/HTL/HIM/perovskite/fullerene (C_60_)/bathocuproine (BCP)/Ag. Different thicknesses of the interfacial materials were tested during the device fabrication, showing that 15 nm of **TACB** and 10 nm of **TTAI** offered better results (Tables S3 and S4). Either PEDOT:PSS or PTAA has been selected as HTL due to its extensive use in PSCs. The active layer is constituted by a triple-cation mixed-halide perovskite with the composition Cs_0.05_(MA_0.15_FA_0.85_)_0.95_Pb(Br_0.15_I_0.85_)_3_, where MA stands for methylammonium and FA for formamidinium. Moreover, devices without HIMs were also fabricated as reference samples. Further details about the device fabrication can be found in the Supporting Information.

**Figure 2:**
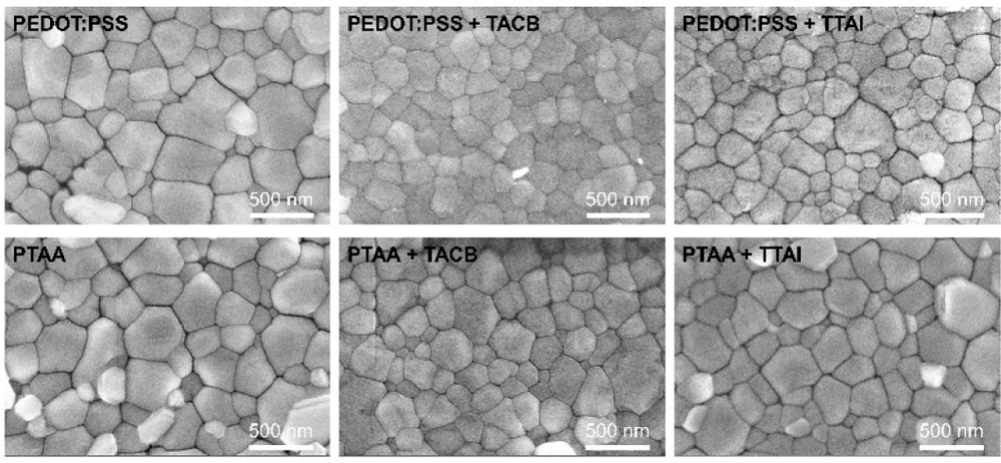
Top-view FESEM images of the photoactive perovskite layers grown on the different HTLs with and without **TACB** and **TTAI** HIMs.

Field emission scanning electron microscopy (FESEM) was employed to investigate the effect of the subjacent material on the morphology of the perovskite layers deposited atop (Figure 2). The FESEM images of the perovskite surface with and without HIMs show compact and uniform films. Nevertheless, the average grain size decreased for the PEDOT:PSS bilayers with **TACB** and **TTAI** (181 and 186 nm, respectively) compared to the simple PEDOT:PSS (242 nm). The average perovskite grain size less significantly decreased from 245 nm for PTAA to 201 and 225 nm for PTAA/**TACB** and PTAA/**TTAI** bilayers, respectively.

**Figure 3:**
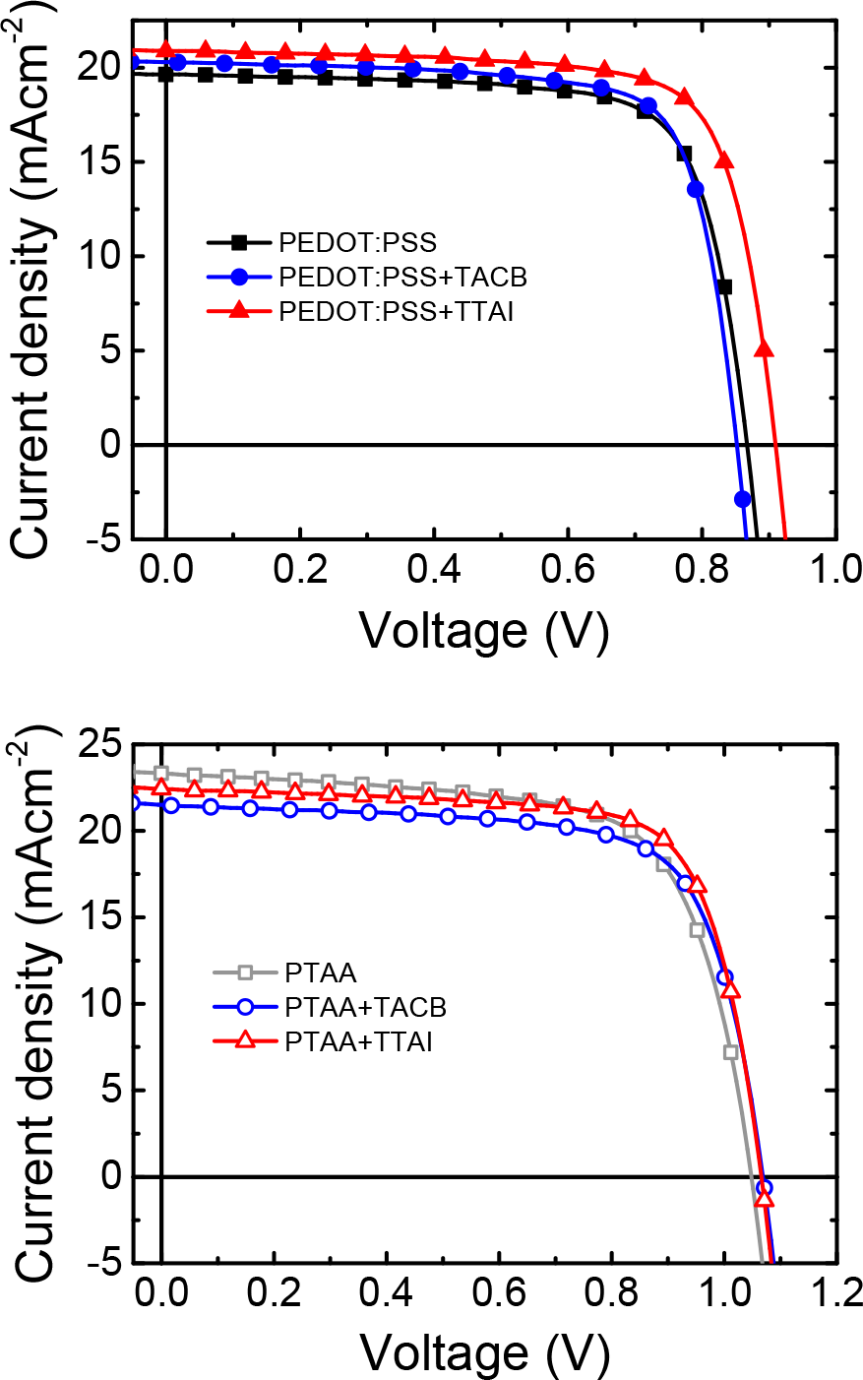
Current density–voltage characteristics under AM1.5 G illumination of the best performing devices.

The current density–voltage curves of the best devices are shown in Figure 3 and Figure S14, while the photovoltaic parameters for those solar cells and the average values for each device are summarized in Table 2. The integration of the HIM combining the PEDOT:PSS/**TACB** HTBL does not noticeably change the average PCE compared to bare PEDOT:PSS. However, the use of the conformationally locked **TTAI** causes a manifest improvement of all the device parameters, see Table 2.

**Table 2:** Device Metrics^a^ Extracted from the *J–V* Characteristics Measured in Different Scan Directions at Simulated AM1.5G Illumination, 100 mW cm^–2^

HTL	scan direction	*J*_SC_ [mA/cm^2^]	*V*_OC_ [V]	FF [%]	PCE [%]	HI^b^
PEDOT:PSS	Reverse	19.64 (19.90 ± 0.38)	0.87 (0.81 ± 0.06)	74.23 (72.87 ± 0.91)	12.64 (11.72 ± 0.90)	0.031
	Forward	19.31 (19.68 ± 0.44)	0.83 (0.78 ± 0.04)	73.38 (73.01 ± 0.70)	11.80 (11.27 ± 0.54)	
PEDOT:PSS/**TACB**	Reverse	20.29 (19.03 ± 0.58)	0.85 (0.80 ± 0.05)	74.86 (75.51 ± 1.47)	12.94 (11.56 ± 1.01)	0.033
	Forward	19.95 (18.86 ± 0.53)	0.84 (0.80 ± 0.04)	74.47 (74.72 ± 0.92)	12.55 (11.26 ± 0.90)	
PEDOT:PSS/**TTAI**	Reverse	20.89 (21.29 ± 0.32)	0.91 (0.87 ± 0.05)	74.83 (73.47 ± 0.84)	14.22 (13.66 ± 0.78)	0.022
	Forward	20.67 (21.05 ± 0.30)	0.89 (0.85 ± 0.04)	74.20 (73.69 ± 0.66)	13.69 (13.20 ± 0.61)	
PTAA	Reverse	22.55 (21.40 ± 0.67)	1.04 (1.02 ± 0.01)	65.33 (65.46 ± 2.18)	15.31 (14.34 ± 0.78)	0.026
	Forward	23.32 (22.97 ± 0.94)	1.05 (1.04 ± 0.01)	68.18 (67.02 ± 1.40)	16.67 (15.96 ± 0.69)	
PTAA/**TACB**	Reverse	20.66 (19.87 ± 0.54)	1.07 (1.04 ± 0.02)	71.35 (68.95 ± 3.97)	15.85 (14.23 ± 1.13)	0.019
	Forward	21.52 (21.06 ± 0.82)	1.07 (1.05 ± 0.01)	71.18 (69.55 ± 3.60)	16.36 (15.31 ± 0.72)	
PTAA/**TTAI**	Reverse	22.21 (21.24 ± 1.36)	1.07 (1.04 ± 0.03)	70.28 (66.68 ± 4.84)	16.66 (14.87 ± 2.16)	0.006
	Forward	22.44 (22.05 ± 0.68)	1.07 (1.05 ± 0.03)	72.95 (68.33 ± 6.42)	17.44 (15.82 ± 2.15)	

a Average values and standard deviation are given in parentheses.

b Hysteresis index.^62^

PTAA-based solar cells present higher performance than PEDOT:PSS, in agreement with previous literature.^62^ On average, samples with a simple PTAA HTL present higher PCE, short circuit current density (*J*_SC_), and open circuit voltage (*V*_OC_) but a lower fill factor (FF) than PEDOT:PSS reference devices. Interestingly, the PTAA/HIM bilayer induces, in general, a FF and *V*_OC_ enhancement but a *J*_SC_ decrease. The lower *J*_SC_ more clearly affects the performance of the PTAA/**TACB** combination. Conversely, the effect on *J*_SC_ is not significant for the conformationally rigid **TTAI**. As a result, the PTAA/**TTAI** combination performs better than bare PTAA. Note that the different interfacial properties of PEDOT:PSS and PTAA are evidenced by the standard hysteresis observed for the former while inverted hysteresis for the latter, with higher apparent device performances for the forward scan. This behavior has been attributed to a different interface polarization.^63^ In this regard, it is worth highlighting that, when fabricating the devices with HTBLs, the hysteresis index (HI)^64^ is perceptively reduced whether **TACB** or **TTAI** is used, particularly in combination with PTAA. Thus, a beneficial interfacial modification has been proved by the incorporation of the HIMs.

The photocurrent densities determined from the integration of the external quantum efficiency (EQE) spectra (Figure S14b) are in good agreement, within the accepted deviation,^65^ with those obtained in the *J–V* curves. These spectra clearly show the increase in the *J*_SC_ for the PEDOT:PSS-based bilayers and its decrease for the PTAA-based bilayers that were previously observed; these *J*_SC_ variations presumably arise from the different EQEs measured within the range between 400 and 600 nm (Figure S14).

**Figure 4:**
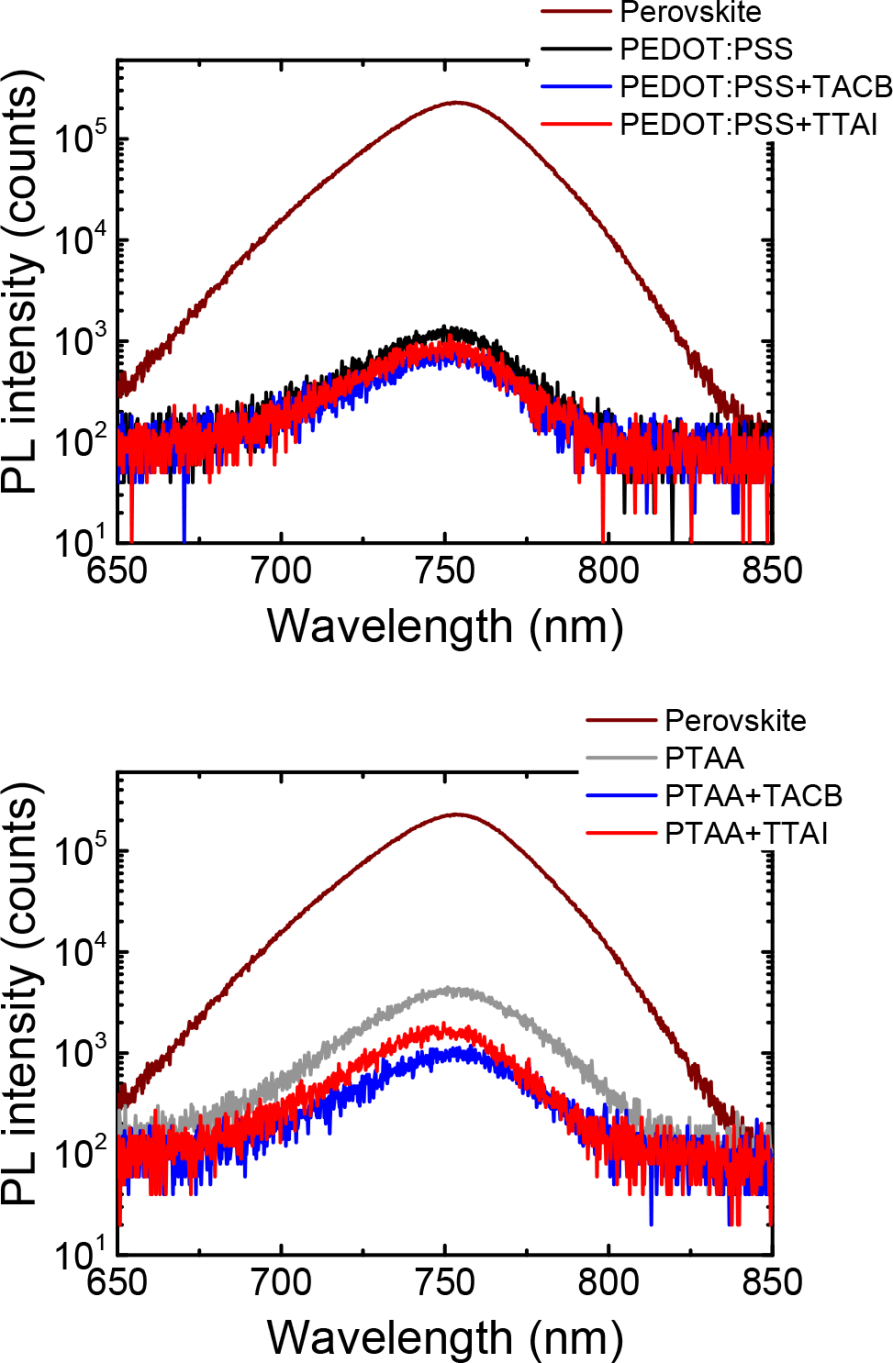
Steady-state PL spectra in semilogarithmic scale of the perovskite film on glass and on the evaluated hole transporting layers.

With the aim of gaining a deeper understanding about the charge extraction at the perovskite interface, steady-state photoluminescence (PL) spectra of the perovskite films deposited onto simple HTLs or HTL/HIMs bilayers were measured. As can be observed in Figure 4, the shape and position of the PL peak, located at ∼755 nm, remained unchanged independently on the underlying material, thus proving that the slight morphological differences previously observed in the FESEM images do not affect the energy of the emissive transition in the perovskite. Nevertheless, a significant decrease of the active layer PL intensity was induced when a hole transporting material was placed underneath. In particular, the incorporation of the small molecular HIMs induced a larger PL quenching than the control films including PEDOT:PSS or PTAA only. This behavior could be correlated to an improved charge carrier transfer from the photoactive perovskite to the underlying interfacial layer, which is favored by the presence of **TACB** or **TTAI**. It is worth noticing that the interpretation of this phenomenon in terms of an increased surface recombination could be discarded given the higher *V*_OC_ values of the small molecular based devices in comparison to the control ones.

**Figure 5:**
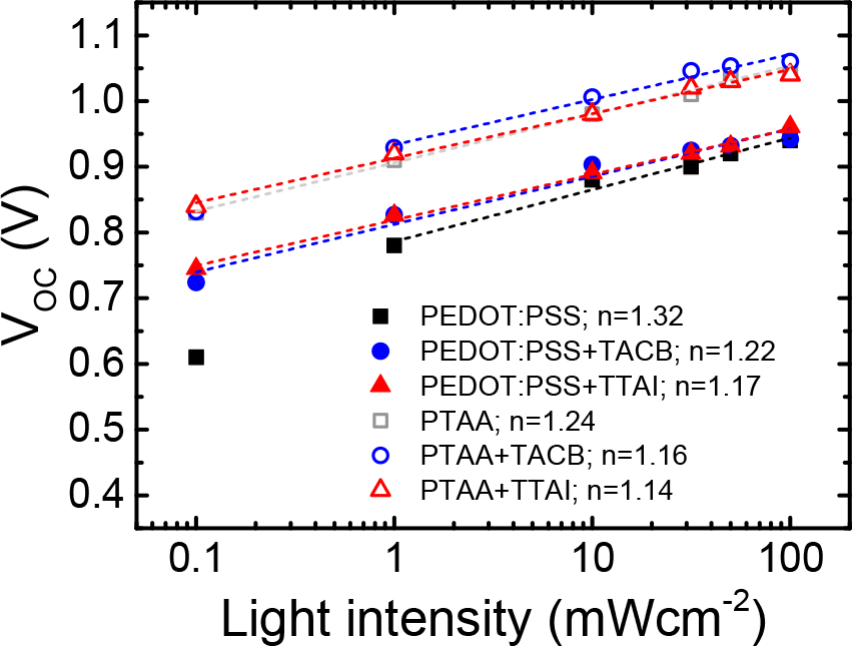
Light intensity dependence of the open-circuit voltage in semilogarithmic scale. Dashed lines represent the linear fits. Ideality factors are shown in the legend.

To investigate the dominant recombination mechanism taking place in the solar cells with the different hole transporting layers, the evolution of the open circuit voltage with the increase of the incident light intensity (*I*) was also analyzed (Figure 5). The representation of the *V*_OC_ values as a function of the light intensity is shown in Figure 5. Light ideality factors (*n*_l_) were estimated from the slope of these plots by fitting to the equation:^66^
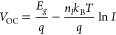
1where *k*_B_ denotes the Boltzmann constant, *T* the absolute temperature, and *q* the elementary charge. The calculated light ideality factors are listed in the legend of Figure 5. In general, an ideality factor of 1 is correlated to a predominant band-to-band radiative recombination. Conversely, a value of 2 is associated to a larger contribution of trap-assisted Shockley-Read-Hall (SRH) recombination.^66^ In both series of devices, lower ideality factor values were obtained after the incorporation of HIMs. Values closer to unity are assessed for **TTAI**, which demonstrate that the incorporation of this rigid, tripodal, self-assembled material contributes to the reduction of the undesired trap-mediated recombination pathways in the device.

**Figure 6:**
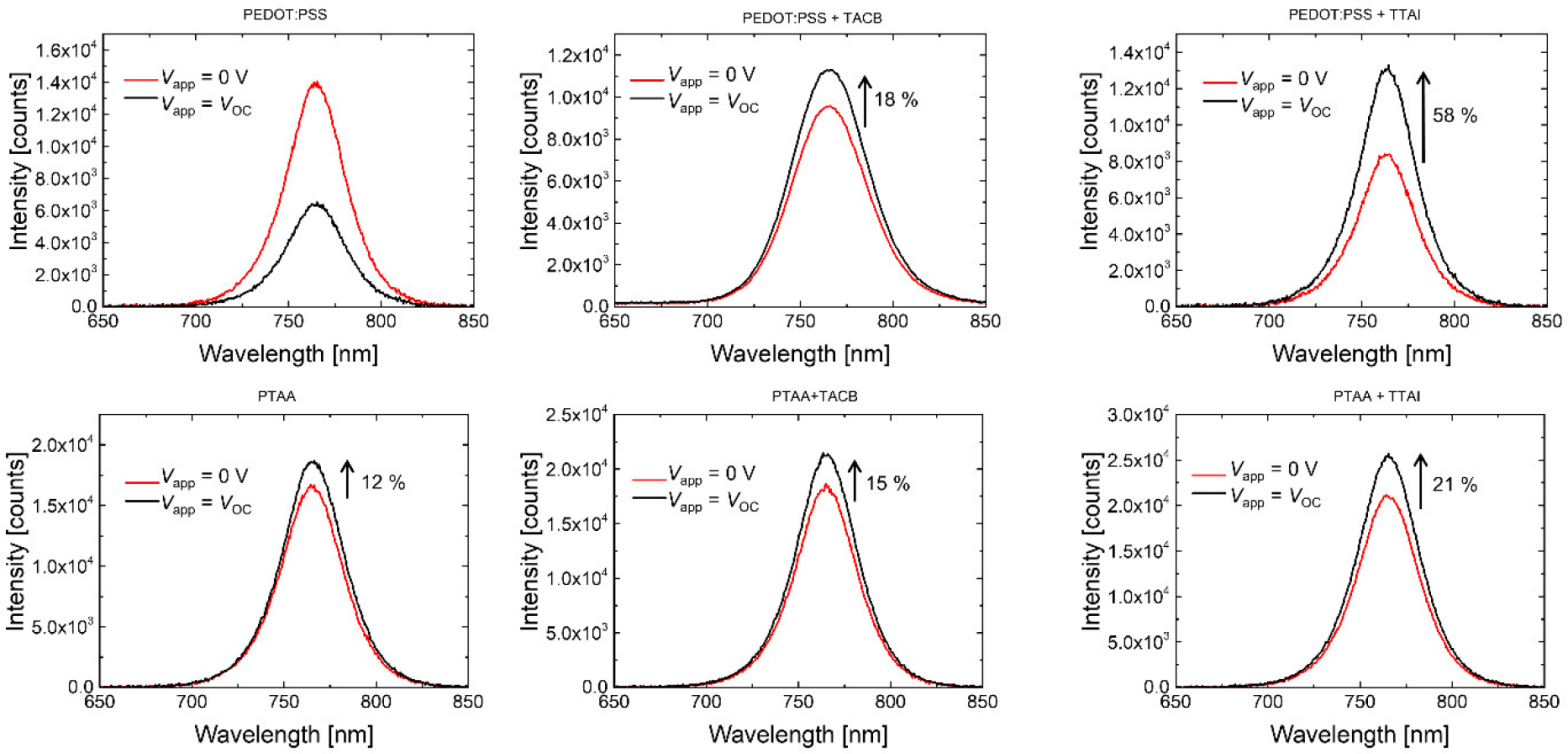
Comparison of the perovskite PL intensity at the active areas of the devices biased at short-circuit (*V* = 0 V) and at *V*_OC_. The PL enhancement ratio is indicated.

The charge extraction and recombination processes occurring at the interface between the perovskite and the charge transport layers have been further investigated by studying the dependence of the PL emitted by the complete solar cells with the applied voltage.^67−70^ When devices are biased at short-circuit conditions (*V*_app._ = 0 V), charge extraction is promoted. Then a better charge extraction would result in a lower PL emission. Upon increasing the applied voltage (*V*_app._ = *V*_OC_), charge extraction is limited and PL is expected to enhance. Moreover, the radiative enhancement will be more significant when nonradiative recombination is reduced. This trend is observed for the different hole transporting layers and bilayers (Figure 6), except simple PEDOT:PSS, presumably due to its known restricted ability to block electrons^71,72^ and, therefore, to minimize the nonradiative charge recombination. The PL enhancement ratio is improved in the devices incorporating HTL/HIM bilayers, being better in the case of **TTAI**. Accordingly, these results seem to indicate that a reduced nonradiative recombination can be achieved by inserting effective HIMs.

Focusing on the devices incorporating **TTAI**, their better performance was further demonstrated by measuring the electroluminescence (EL) of the PSCs (Figure S15). Larger EL intensity was observed when HTL/**TTAI** bilayer was present in comparison to the PEDOT:PSS and PTAA single HTLs. These results clearly reinforce that the HIMs promote a reduction of the nonradiative recombination pathways within the device, which is in good agreement with the previously discussed PL experiments.

**Figure 7:**
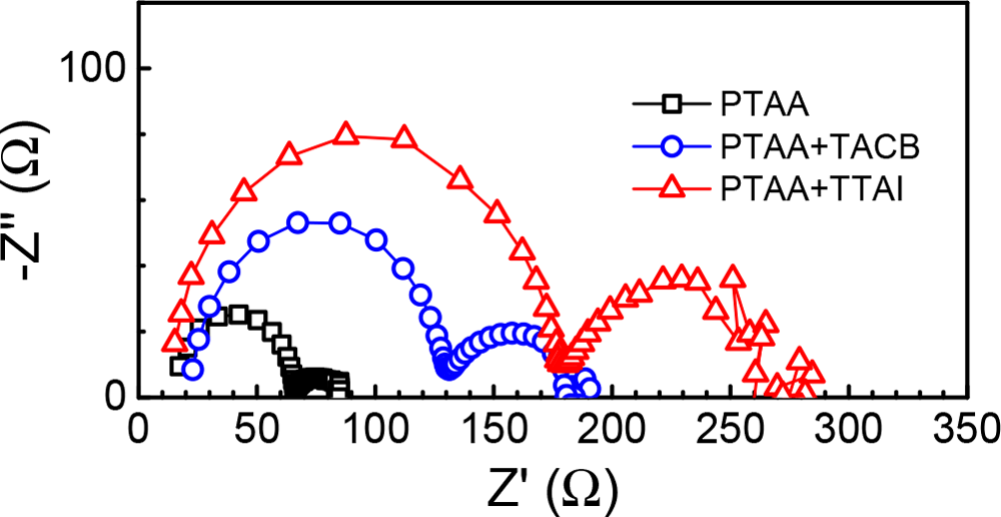
Nyquist plot at *V*_OC_ for a PTAA single HTL, PTAA/**TACB**, and PTAA/**TTAI** hole transport bilayer.

Finally, to complete the electrical characterization and understand the effect of 2D-self-assembled π-expanded tripodal molecules (**TACB** and **TTAI**) as interfacial materials, electrochemical impedance spectroscopy (EIS) measurements were performed under 1 sun illumination (voltage range 0 to *V*_OC_). The EIS experimental details and the equivalent circuit employed to analyze the data are detailed in the Supporting Information. The acquired impedance data were fitted to an equivalent circuit model including a recombination resistance (*R*_rec_) considering the transport resistance negligible, and a series resistance (*R*_s_).^73^ Focusing on the best performing devices fabricated with PTAA, the corresponding Nyquist plots exhibited two distinctive arcs located in the low- and high-frequency regions (Figure 7). In agreement with the Z′ values, it is observed that the recombination resistance increases when introducing both **TACB** and **TTAI**, with respect to the reference device made with PTAA only. Accordingly, the device fabricated with **TTAI** presents a lower carrier recombination and, consequently, a higher *V*_OC_ and *FF* as shown in Table 2.

This enhancement emphasizes the relevance of the structure–property relationship showing how, having **TACB** and **TTAI** similar optoelectronic properties, the conformationally locked **TTAI** presents suitable structural features for better performance in the studied hole transport bilayers.

## Conclusions

In conclusion, two unprecedented small molecular materials with analogous structure but different conformational freedom have been synthesized. The tripodal skeleton of these molecules has been rationally designed to incorporate hydrogen bond donor and hydrogen bond acceptor sites that induce the 2D-self-assembly of these materials. Self-assembly has proved to be a useful tool to improve the robustness of organic thin films. Both molecules, **TACB** and **TTAI**, display a suitable electronic structure to be integrated as hole transporting interfacial materials in perovskite solar cells. This has been demonstrated by the improvement in the performance of the solar cells when combining the novel materials with standard HTLs such as PEDOT:PSS and PTAA, reaching a 17.4% efficiency. The engineering of the hole transporting bilayers, with an adequate energy gradient and intermolecular interactions at the perovskite interface, has led to the reduction of the recombination pathways and the improvement of hole extraction at the perovskite interface. Overall, we can conclude that the strategy based on the self-assembly of interfacial materials such as **TTAI** is a valid approach for the future development of carrier selective transport layers.

## Supporting Information Available

NMR spectra; TGA and DSC; computational details; X-ray diffraction of **TACB**; cyclic voltammetry, energy levels diagram, current density vs voltage curves and external quantum efficiency spectra; devices electroluminescence (PDF)
CIF file of TACB crystal structure (CIF)

## Supplementary Material

am2c23010_si_001.pdf

am2c23010_si_002.cif
