# Supplementary material for: 2D-Self-Assembled Organic Materials in Undoped Hole Transport Bilayers for Efficient Inverted Perovskite Solar Cells
Source: ACS Appl Mater Interfaces. 2023 Apr 26;15(18):22310–9. doi: 10.1021/acsami.2c23010 (PMC10176319; doi:10.1021/acsami.2c23010)
Supplement: Supplementary file 1 — am2c23010_si_001.pdf [file am2c23010_si_001.pdf]

SUPPORTING INFORMATION

2D-Self-Assembled Organic Materials in Undoped Hole Transport Bilayers for Efficient Inverted Perovskite Solar Cells.

Isaac G. Sonsona,[a] Manuel Carrera,[a] Miriam Más-Montoya,[a,*] Rafael S. Sánchez,[b] Patricio Serafini,[b] Eva M. Barea,[b] Iván Mora-Seró [b,*] and David Curiel [a,*]

[a] Department of Organic Chemistry, Faculty of Chemistry, University of Murcia, 3100-Murcia, Spain.

[b] Institute of Advanced Materials, University Jaume I, Avenida de Vicent Sos Baynat, s/n, 12071 Castelló de la Plana, Spain.

Miriam Más-Montoya: miriammas@um.es

Iván Mora-Seró: sero@uji.es

David Curiel: davidcc@um.es

1.-General S2

2.-NMR spectra S3

3.-Thermal characterization S6

4.-Computational details S7

5.-X-ray diffraction S10

6.-Cyclic voltammetry S11

7.-Energy levels diagram S11

8.-Single carrier devices S12

9.-Current density vs. voltage curves and External Quantum Efficiency spectra S13

10.-Devices electroluminescence S15

11.-References S15

1.-General

Charge transport measurements

Hole only devices with the architecture: ITO/MoO_3 (10 nm)/TACB (80 nm) or TTAI (100 nm) /MoO_3 (10 nm)/Ag (100 nm) were fabricated by thermal evaporation under high vacuum conditions. Current density versus voltage curves were measured using a Keithley 2636A SMU. These curves were fitted to the Murgatroyd equation: $J = (9/8)\varepsilon_0\varepsilon_r\mu_0(V^2/L^3)\exp[0.89\,\beta(V/L)^{1/2}]$, where J is the current density, ε_0 the vacuum permittivity (8.85×10^{-14} F cm^{-1}), ε_r the relative permittivity of the organic layer (≈3), μ_0 the zero-field mobility, L the thickness of the organic layer, and β the field-activation factor. The hole mobility was determined using the Poole-Frenkel relationship, $\mu=\mu_0\exp(\beta E^{1/2})$, at an electric field of 10^5 V cm^{-1}.

Solar cells fabrication and characterization.

Pre-patterned ITO glass substrates were cleaned by sequential sonication in an Extran aqueous solution, deionized water, ethanol, acetone, and isopropanol for 15 minutes each step. Right before using, the substrates were subject to an UV-ozone treatment for 30 minutes. Then, the hole transporting materials were deposited. PEDOT:PSS (Heraeus, Clevios PVP AI 4083) filtered with a 0.45 μm PVDF filter was spin coated at 5000 rpm for 40 seconds under ambient conditions. The substrates were then annealed at 130 °C for 20 minutes. A 2 mg/mL solution of PTAA in toluene was spin coated at 5000 rpm for 30 seconds in a nitrogen-filled glovebox and the substrates were annealed at 100 °C for 10 minutes. Those devices incorporating the small molecular materials were transferred to a vacuum chamber to deposit a thin layer of about 10 nm by thermal evaporation under high vacuum (~10^{-7} mbar). The perovskite active layer was prepared in a nitrogen-filled glovebox with the composition $Cs_{0.05}(MA_{0.15}FA_{0.85})_{0.95}Pb(Br_{0.15}I_{0.85})_3$. The perovskite precursor solution was prepared by mixing in a 85:15 volume ratio a formamidium iodide solution (1.24 M), dissolved in a solution of PbI_2 (1.5 M) in DMF:DMSO (4:1 volume ratio), and a methylamonium bromide solution (1.24 M), dissolved in a solution of $PbBr_2$ (1.5 M) in DMF:DMSO (4:1 volume ratio). The resulting solution was mixed with CsI (1.5 M) dissolved in DMSO in a 0.95:0.05 volume ratio. The freshly prepared precursor solution was deposited on the HTL-coated substrates at 4000 rpm for 30 seconds. After 25 seconds, 250 μL of chlorobenzene was dropped onto the spinning substrate. Subsequently, an annealing step was performed at 100 °C for 30 minutes. The devices were finished by the thermal evaporation under high vacuum conditions (~10^{-7} mbar) of a 23 nm thick layer of C60, a 8 nm thick layer of bathocuproine, and a 100 nm thick Ag top electrode. The current-voltage curves were measured using a Keithley 2612 source meter under AM 1.5 G (1000 Wm^{-2}) in a Solar Simulator Abet, Xenon short-arc lamp Ushio 150 watts, in air at room temperature. A scan rate of 10 mVs^{-1} was used. External Quantum Efficiency measurements were carried out using a QEPVSI-b Oriel system also in air.

Photoluminescence measurements of complete devices at different potentials

The photoluminescence of the complete devices at different applied potentials were recorded on a homemade setup that consists of a 532 nm solid state laser diode (5 mW, 0.0314 cm^{-2} beam spot) as an optical excitation source, a CCD detector (Andor-iDUS DV420A-OE) coupled to a spectrograph (Kymera-193I-B2) setup for the optical detection, and a potentiostat (GAMRY Reference 3000) for electrical biasing.

Electrochemical Impedance Spectroscopy (EIS)

Measurements were carried out on complete devices performed by applying 20 mV of a voltage perturbation at different frequencies from 1MHz to 0.1Hz in a PGSTAT-30 from Autolab and under 1 sun illumination at different applied forward biased, from 0 to 1.1 volts (up to V_{OC} value). The equivalent circuit used to fitting the measurements it is shown below,[1] where transport resistance has been neglected.

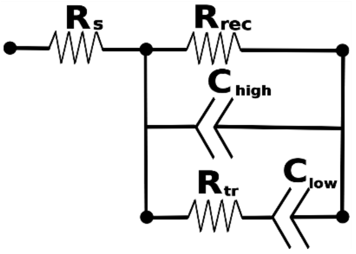

Morphology

Field Emission Scanning Electron Microscopy images of the samples mounted on aluminum stubs were examined using a FE-SEM (ApreoS Lovac IML Thermofisher) with a selected voltage of 5 kV and 0.10 nA under high vacuum conditions. T3 detector and immersion mode were used for sample imaging.

2.-NMR spectra

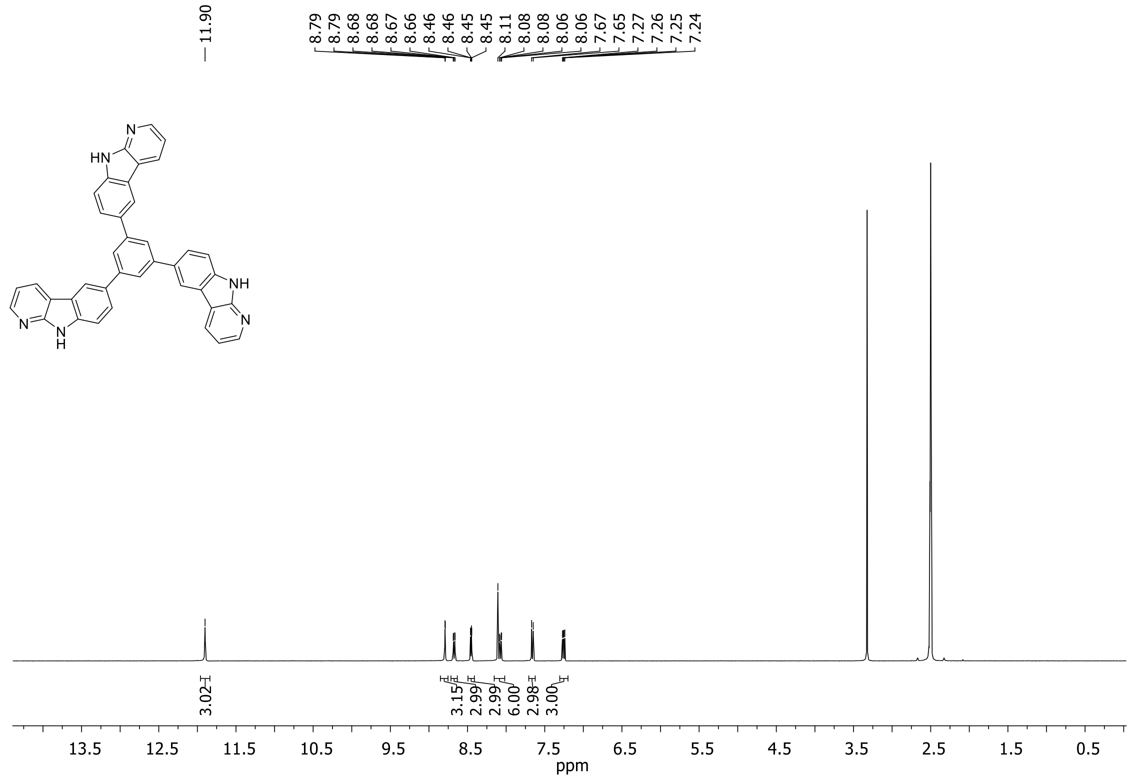

Figure S1. ^{1}H-NMR spectrum of **TACB** (400 MHz, DMSO-d_6).

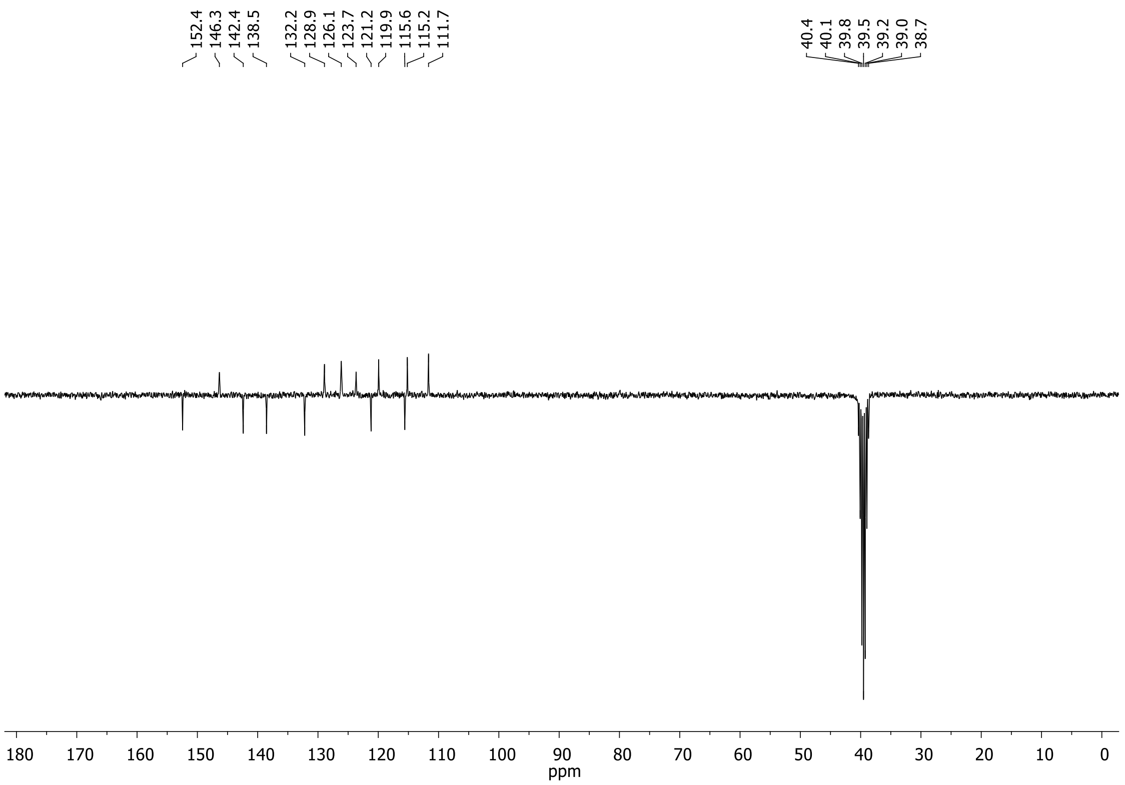

Figure S2. ^{13}C-NMR(APT) spectrum of compound **TACB** (75.5 MHz, DMSO-d_6).

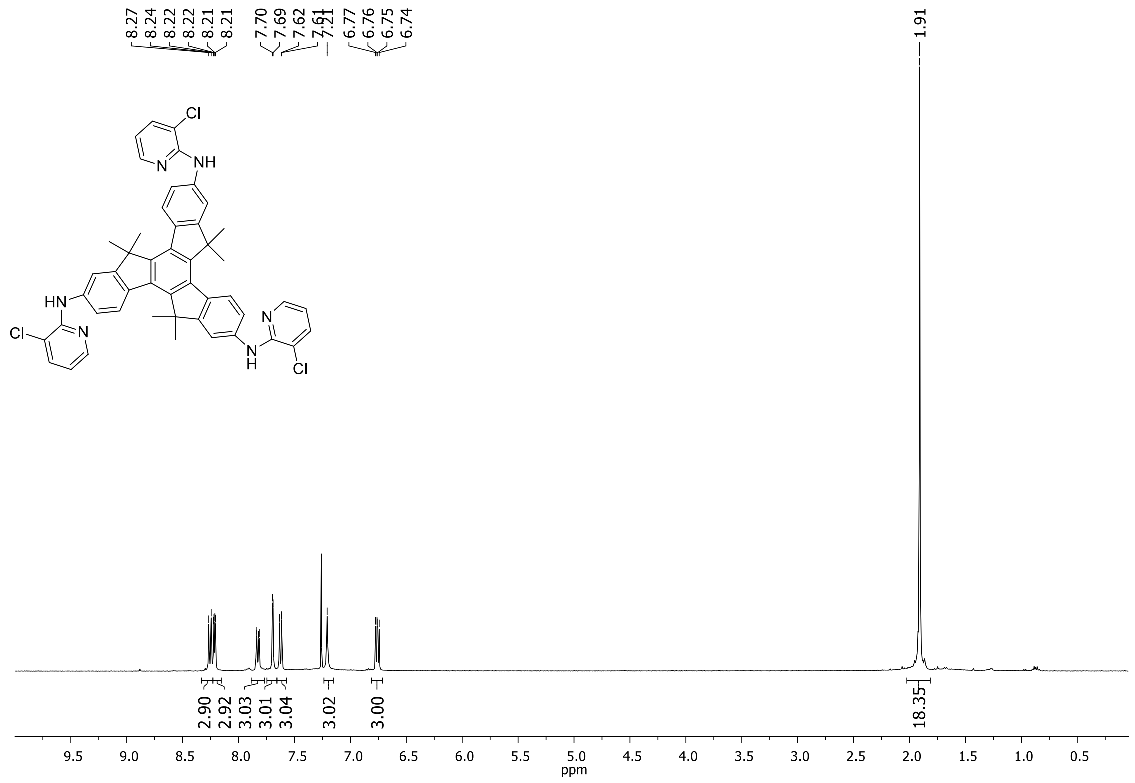

Figure S3. ^{1}H-NMR spectrum of compound **4** (400 MHz, C*D*Cl$_3$).

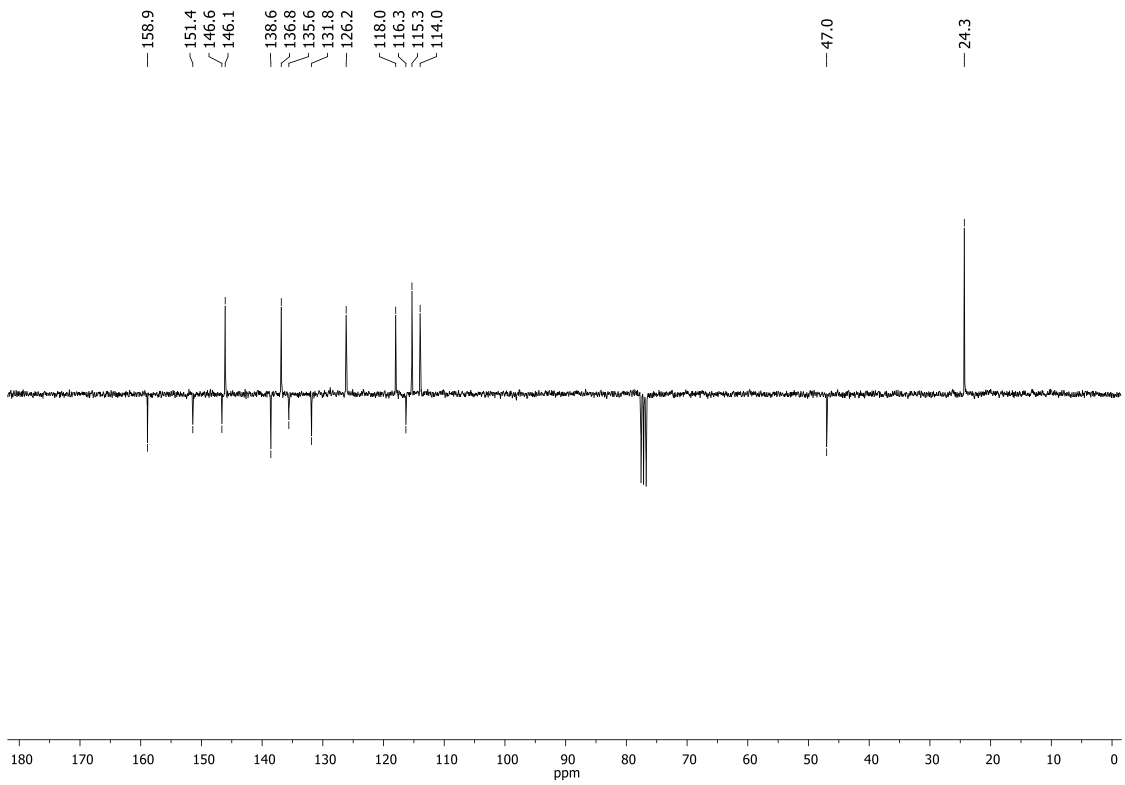

Figure S4. ^{13}C-NMR(APT) spectrum of compound **4** (75.5 MHz, C*D*Cl$_3$).

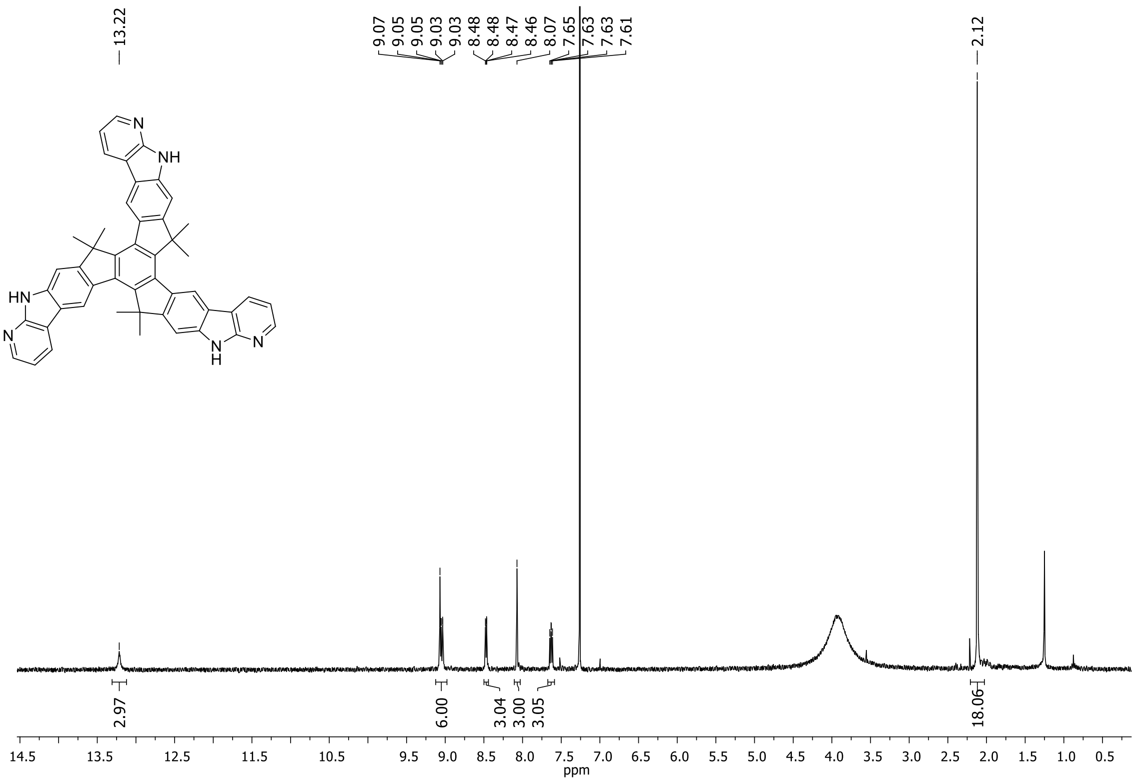

Figure S5. ^{1}H-NMR spectrum of **TTAI** (400 MHz, $CDCl_3$ (1% TFA-*d*)).

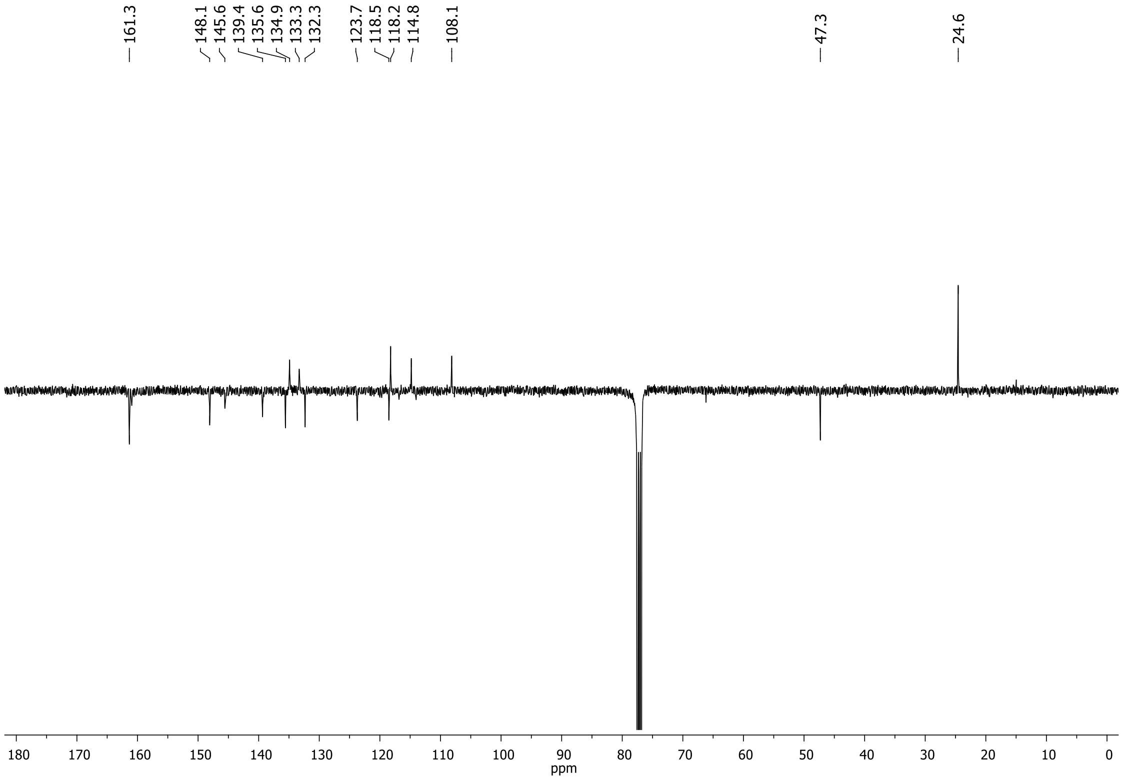

Figure S6. ^{13}C-NMR(APT) spectrum of **TTAI** (75.5 MHz, C*D*Cl_3 (1% TFA-*d*)).

3.-Thermal characterization

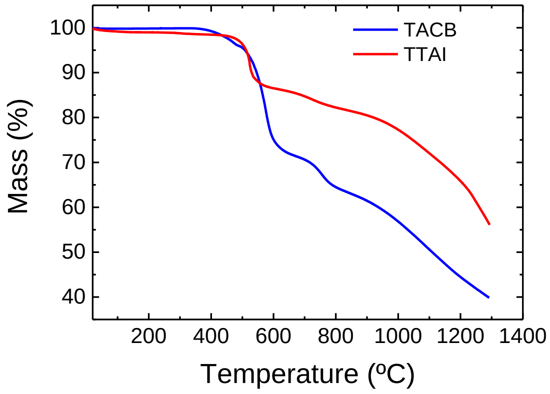

Figure S7. Thermogravimetric Analysis (TGA).

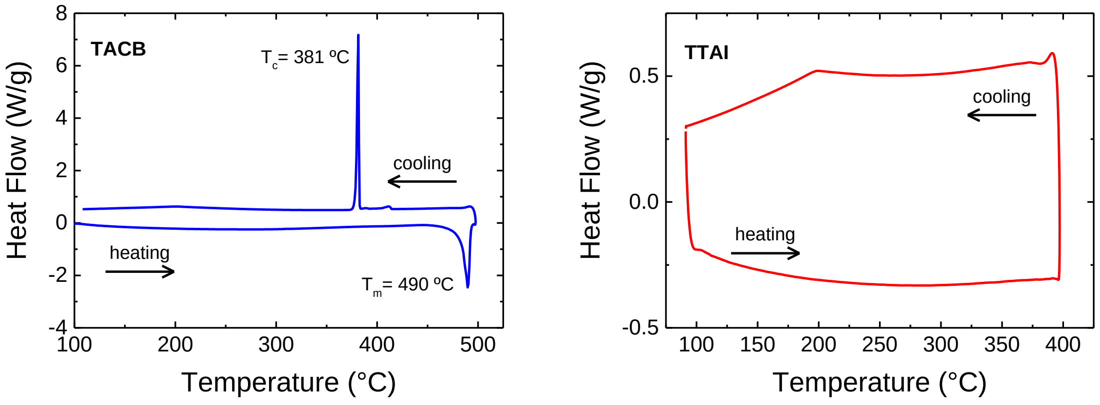

Figure S8. Differential Scanning Calorimetry (DSC).

4.-Computational details

Geometry optimizations were carried out by DFT calculations using the ORCA program, being run in redundant internal coordinates with tight convergence criteria.[2] All calculations were performed using the B3LYP functional,[3] the def2-TZVP basis set,[4] the RIJCOSX algorithm,[5] and dispersion corrections (DFT-D4).[6] All geometry optimizations took into account the effect of the solvent (dimethylformamide) by means of the CPCM model.[7]

Table S1. Computed structure for **TACB** (CPCM(DMF)/B3LYP-D4/def2-TZVP level)

C	0.78466875519828	3.87683819519645	1.06026079149116
C	0.62438987610831	5.27507406416905	0.96970362288622
C	1.69676029796585	6.13471894562723	0.79731850511583
C	2.96677426837605	5.57605359140123	0.70934031856359
C	3.16119809934135	4.17706410205621	0.80277995448994
C	2.06690230529728	3.33590255893014	0.98022454903078
N	4.18933378522390	6.18982394804833	0.52636231503973
C	5.17322065718341	5.23347642609464	0.49155762996692
C	4.57873852509013	3.95472634768056	0.66196617847001
N	6.46651444450019	5.47356111206258	0.32251329860654
C	7.24980999272899	4.38754646688179	0.31952279737309
C	6.78220121932876	3.08217966820707	0.48093326349614
C	5.42104561034686	2.85258196390350	0.65556975113344
C	-0.32801085153606	1.84806285521776	2.01196423774117
C	-0.39588513830327	2.99923351865784	1.22480774420458
C	-1.59950803058213	3.30201259620342	0.58600123535843
C	-2.72088558212312	2.48217676957945	0.72208914387418
C	-2.62257277159156	1.33786920297754	1.51509091323827
C	-1.43136401488113	1.00657791524828	2.16379554573321
C	-3.98656623296426	2.82173913933473	0.03415713144463
C	-5.22429702360009	2.61632637056069	0.67709039866851
C	-6.43235863540369	2.92787471342767	0.07419258090890
C	-6.39754393657322	3.45265219092910	-1.21292704360457
C	-5.17066864824295	3.66512594242502	-1.88625385380712
C	-3.97102640884901	3.35114226181433	-1.25504999143888
N	-7.43564853971601	3.83631666903513	-2.03816892475346
C	-6.92313820870159	4.28328102673946	-3.23032430599224
C	-5.50632568147588	4.19459185180298	-3.18450120665768
N	-7.64205021281095	4.72118754874102	-4.25519015697730
C	-6.93216358469887	5.10182744871858	-5.32485217121687
C	-5.53883861743448	5.05875297395833	-5.39827069871630
C	-4.80364627913330	4.59813616373704	-4.31038508349757
C	-1.33615725695326	-0.21873511913635	2.98876731686284

C	-0.59694240049907	-0.21253761179950	4.18951475790114
C	-0.47407434672283	-1.33665593698452	4.98985199814477
C	-1.10548502744478	-2.50227665740609	4.57043223876507
C	-1.85336495425291	-2.54028968838184	3.36906528990889
C	-1.96687149051920	-1.39496670459917	2.58693641585324
N	-1.13916450827804	-3.74943966875735	5.16134269608164
C	-1.88013203885652	-4.59938916765098	4.37891270492259
C	-2.34860783508518	-3.88828149735113	3.24210035577221
N	-2.11403226749576	-5.87687406548253	4.64769883711249
C	-2.86200835944272	-6.51979956446744	3.74215340973251
C	-3.37728093535697	-5.92851193411234	2.58733055556230
C	-3.11965154531019	-4.58659780851950	2.32452719720802
H	-0.36900200073218	5.69547037684913	1.05448902170849
H	1.54880220971787	7.20521484897139	0.73812223527747
H	2.20989131400404	2.26414511757469	1.03086884887191
H	4.34452923115283	7.17925282402610	0.42070378040099
H	8.31028076789822	4.56600415556206	0.18037617120177
H	7.48567130636601	2.26095141712786	0.46766845224595
H	5.03761112399377	1.84755444426537	0.78064562824285
H	0.59871016292953	1.60205592462762	2.51276812460758
H	-1.66666892144150	4.19053480939853	-0.02712071464693
H	-3.48484703695094	0.69432216366923	1.62804936382843
H	-5.23212754160491	2.22125205359451	1.68459193970001
H	-7.36989572076779	2.77260233858361	0.59216785184273
H	-3.03097873081483	3.49761712659375	-1.77152394258881
H	-8.41675621869564	3.78916501688297	-1.81585331913794
H	-7.50521051901553	5.46138555754502	-6.17233190309710
H	-5.04451557112043	5.38461374903124	-6.30320662710713
H	-3.72210763735597	4.55651413795976	-4.34689080028891
H	-0.12382951164221	0.70702391398375	4.50835525474362
H	0.09009554480966	-1.30436735415114	5.91291680198266
H	-2.52389134790196	-1.42420061962807	1.65916380818223
H	-0.68558944810373	-4.00942745168951	6.02208464968760
H	-3.06240304430953	-7.56559131260997	3.94714841875948
H	-3.97195967263731	-6.52240747682594	1.90675753815089
H	-3.50859520962776	-4.10886488606046	1.43389717343118

Table S2. Computed structure for **TTAI** (CPCM(DMF)/B3LYP-D4/def2-TZVP level)

C	1.08128582652514	-0.14696511105151	0.06228117598174
C	2.19854392623781	0.68744473948624	0.03787738113780
C	2.03505400111732	2.09299889768721	0.00015032653673
C	0.75403448620629	2.64183628729713	-0.01478665710151
C	-0.38291874133741	1.79994664816945	0.01035707979649
C	-0.21755461910651	0.41663554838240	0.04864550166353
C	0.33848453129685	4.10494377081911	-0.05844299709734
C	-1.16939512223053	3.99971511158919	-0.05950432359387
C	-2.04789413950717	5.06419125542266	-0.09801554689464
C	-3.40450616931344	4.76156293856971	-0.09013182327428
C	-3.86131804223618	3.42271346136975	-0.04242094444684
C	-2.95264054553748	2.36818294044022	-0.00487205544185
C	-1.59040877768051	2.65281539103031	-0.01513074846383
H	-1.70243957957051	6.08949887370295	-0.13351710633666
H	-3.33355578442488	1.36339642411467	0.03045312060814
C	3.67707942263601	0.32056600603462	0.04419316930377
C	4.33568934574653	1.68129050192044	0.00646504266679
C	3.37537993300332	2.71547516030980	-0.01752062591776
C	-1.27833544420695	-0.67411848768450	0.07927412702865
C	-0.43364786966307	-1.92730573276851	0.11032112771876
C	0.94429885045066	-1.61884726601572	0.10095447768511
C	5.69697107157145	1.91548330560523	-0.00262019555331
C	6.10782230187635	3.24310806940879	-0.03519656435901
C	5.17087537776550	4.30372190104465	-0.05805416929679
C	3.80431698289487	4.03891587206796	-0.04949813939092
H	6.41635628453249	1.10676086660496	0.01616819774596
H	3.12085173007257	4.86854068978223	-0.06695137201147

C	0.02200281970454	-4.24375462798966	0.16513009150878
C	1.41082327034024	-3.96993200638175	0.15736981472232
C	1.87160991937502	-2.65612092126332	0.12601128353146
C	-0.91781042693355	-3.21997541442035	0.14241695794289
H	2.93302387471670	-2.48359462774216	0.12152367187717
H	-1.97908833802246	-3.43308951842955	0.14878101018325
C	7.30656646742018	5.14096152119628	-0.07733429182200
C	5.93873247023609	5.52364654457912	-0.08539783754859
N	7.38356485802285	3.77020607718540	-0.04848564238007
H	8.24126762531868	3.24257640813955	-0.03605595562398
C	5.65602102580010	6.88109944871989	-0.11470549605192
C	8.03543885541555	7.26863767677724	-0.12108325439069
C	6.73145186526205	7.76476127152847	-0.13289674680406
H	4.63668891750688	7.24675355695677	-0.12284886616827
H	8.87416450461147	7.95573391709240	-0.13393649331949
H	6.57025978467048	8.83387935192737	-0.15546377553565
N	8.34434115523125	5.96545231655646	-0.09378761108559
C	-5.64687531935711	4.86237874311052	-0.09992193157894
C	-5.30191324734737	3.48511430851991	-0.04789865732222
N	-4.49409870408310	5.60797831592571	-0.12398192585622
H	-4.46030508018786	6.61375238788111	-0.16220010818363
C	-6.34186059570645	2.56781385581969	-0.01702527767107
C	-7.85630883365855	4.44280681363507	-0.09247264321217
C	-7.64180388091411	3.06499230649932	-0.04059168301862
H	-6.15604240021806	1.50163912491374	0.02350514646764
H	-8.86865531347328	4.83079990342058	-0.11059380649048
H	-8.49127198249530	2.39601092697275	-0.01949113724575
N	-6.87679479457159	5.35606700307801	-0.12251165275888
C	1.05465522748565	-6.23620240911485	0.20826030924945
C	2.07586503650502	-5.24885924048356	0.18544686048915
N	-0.16747342118808	-5.61073825616401	0.19622825055154
H	-1.05611740406725	-6.08450275394238	0.20577638390843
C	4.09805139993551	-0.38460753800520	1.34974551976855
H	3.55629169548267	-1.31022305812323	1.52285502033086
H	5.16480462880492	-0.61441890694332	1.32562160789400
H	3.91111531135039	0.27240248252284	2.20082450067126
C	4.09164841738744	-0.45391753000456	-1.22351740067664
H	3.90073607792017	0.15643514052787	-2.10777466412607
H	5.15846454998481	-0.68257703616321	-1.19228890042385
H	3.54893071642477	-1.38726181136530	-1.34408916525808
C	-2.12955736286142	-0.69474555809448	-1.20741592935152
H	-2.87133321005480	-1.49394185194265	-1.15602371047805
H	-2.65201653731891	0.24112960801478	-1.38347044679059
H	-1.48791450460665	-0.88208116603788	-2.07004591391155
C	-2.12374918616797	-0.62568637248985	1.36908816263962
H	-1.47903396309608	-0.77306519707070	2.23715959960019
H	-2.63983473035012	0.32113692326697	1.50012439778149
H	-2.87033740600895	-1.42205349111818	1.36150638437333
C	0.77666534230435	4.79359268654926	-1.36764189741959
H	0.30607835630756	4.29770955925371	-2.21827065156096
H	0.46266766980972	5.83905369886392	-1.36784965318671
H	1.85133655130002	4.76235238723786	-1.52316085321442
C	0.76831781514099	4.86703008376823	1.21274082667388
H	0.29098262076765	4.42092528364785	2.08683479841920
H	1.84178357387979	4.84260591943922	1.37753967789001
H	0.45607356394302	5.91121481155189	1.15156949046534
C	2.52232500700874	-7.94021806099007	0.24346672840830
C	3.60920011851918	-7.06541208096992	0.22334885741875
C	3.39019636189316	-5.69095228560230	0.19372466039166
H	2.69171016906926	-9.01097862737766	0.26629707736287
H	4.61350368840890	-7.46645638838584	0.23093447552251
H	4.22108984836203	-4.99648572440517	0.17795460835818
N	1.24151624394260	-7.54810596739813	0.23627034737088

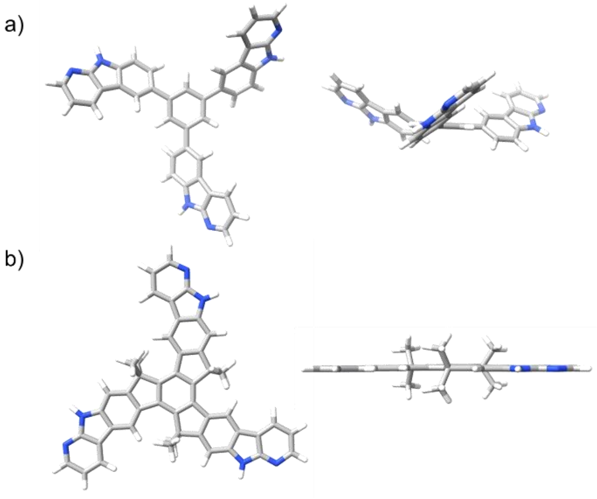

Figure S9. Top and side views of the computationally optimized geometries at the RIJCOSX-B3LYP-D4/def2-TZVP level based on the conductor-like polarizable continuum (CPCM) model for a) **TACB** and b) **TTAI**.

5.-X-ray diffraction

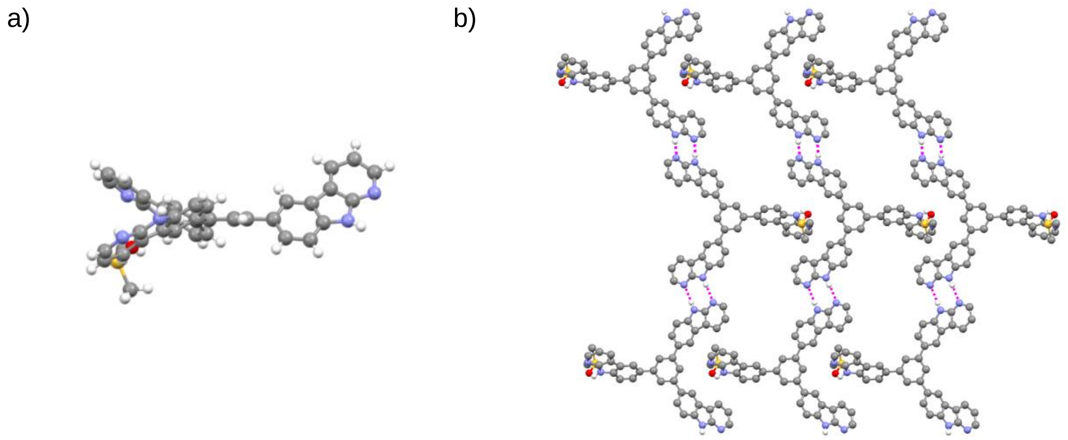

Figure S10. a) Crystal structure of **TACB** including one molecule of crystallization solvent; b) Crystal packing of **TACB** governed by hydrogen bonding. Crystallization solvent interferes with the conformational arrangement of the tripodal **TACB** molecule.

6.-Cyclic voltammetry

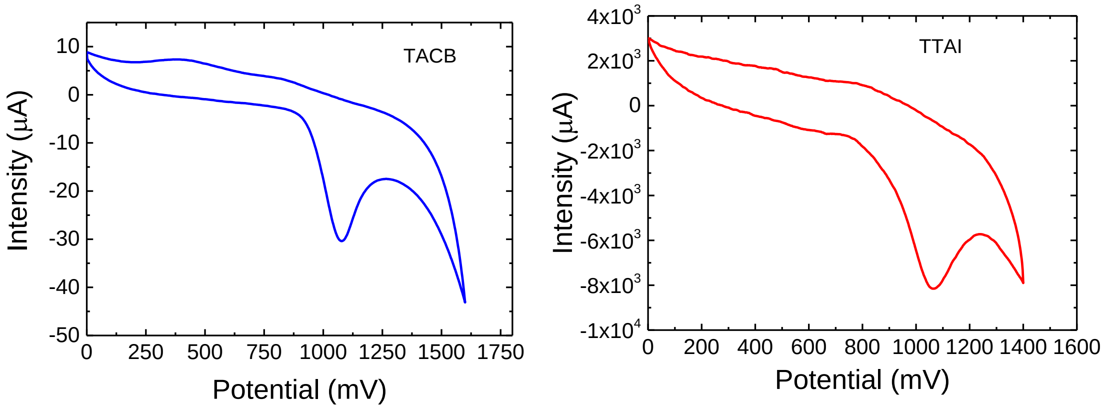

Figure S11. Cyclic voltammograms of a thin film drop casted on a platinum working electrode in dichloromethane; reference electrode: Ag/AgCl; counter electrode: platinum wire; supporting electrolyte: TBAPF6; scan rate: 100 mVs^{-1}; internal reference: ferrocene/ferrocinium.

7.-Energy levels diagram

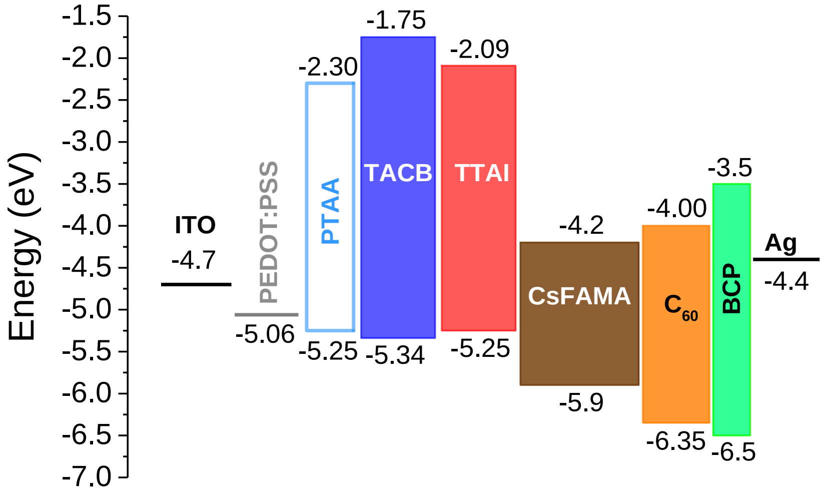

Figure S12. Energy level diagram.[8]

8.-Single carrier devices

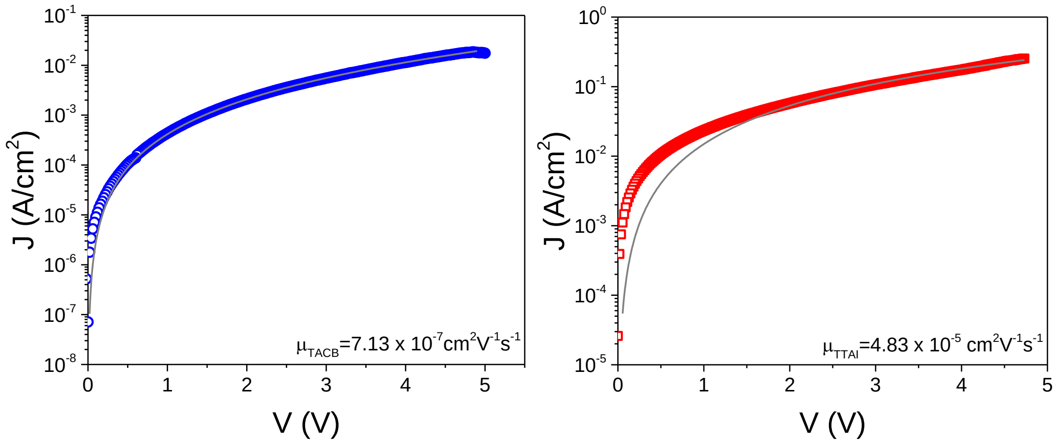

Figure S13. *J-V* characteristics of the **TACB** (left) and **TTAI** (right) hole-only devices. The solid line represents the fit to the Murgatroyd equation with a field-dependent mobility.

9.-Current density vs. voltage curves and External Quantum Efficiency spectra

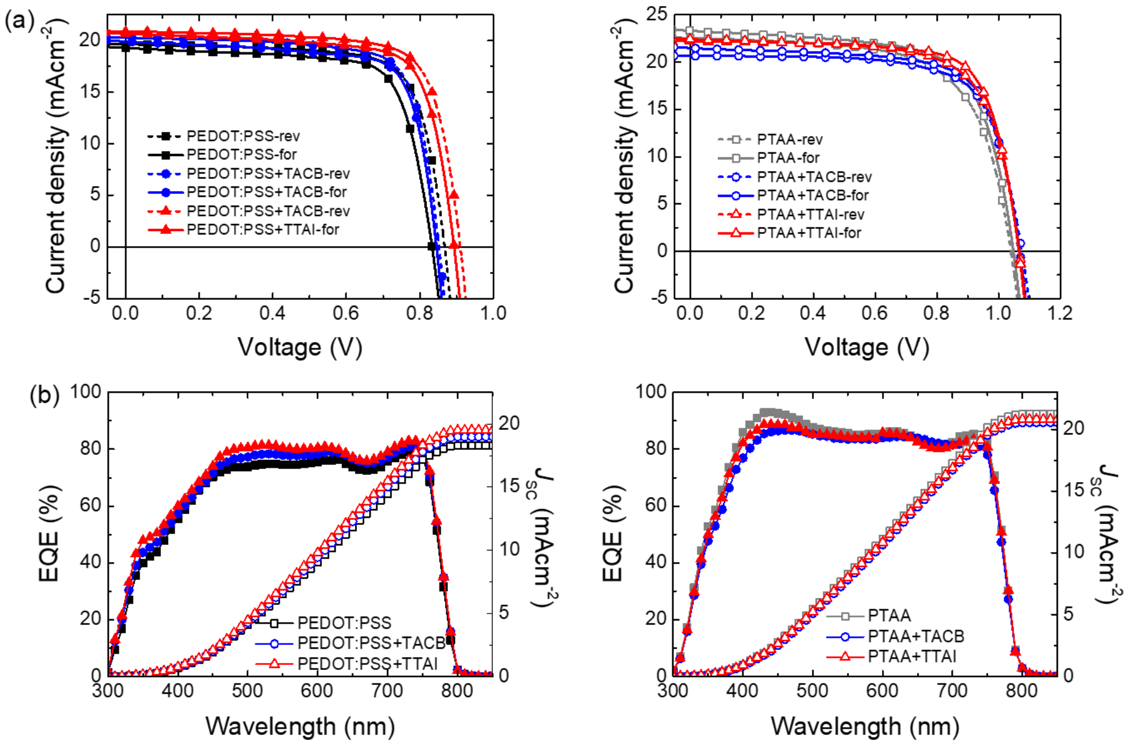

Figure S14. (a) Forward and reverse current density-voltage characteristics under AM1.5 illumination; (b) External Quantum Efficiency (EQE) spectra.

Table S3. Photovoltaic parameters at different HIM thicknesses for PEDOT:PSS/HIM bilayers.

HTL	HIM	Scan direction	J_{SC} [mA/cm^2]	V_{OC} [V]	FF [%]	PCE [%]
PEDOT:PSS		reverse	19.64 (19.90±0.38)	0.87 (0.81±0.06)	74.23 (72.87±0.91)	12.64 (11.72±0.90)
		forward	19.31 (19.68±0.44)	0.83 (0.78±0.04)	73.38 (73.01±0.70)	11.80 (11.27±0.54)
PEDOT:PSS	TACB 10 nm	reverse	20.55 (19.95±0.66)	0.92 (0.87±0.04)	64.58 (65.61±5.52)	12.20 (11.37±0.68)
		forward	20.26 (19.69±0.76)	0.91 (0.86±0.04)	65.90 (67.60±2.76)	12.13 (11.47±0.42)
PEDOT:PSS	TACB 15 nm	reverse	20.29 (19.03±0.58)	0.85 (0.80±0.05)	74.86 (75.51±1.47)	12.94 (11.56±1.01)
		forward	19.95 (18.86±0.53)	0.84 (0.80±0.04)	74.47 (74.72±0.92)	12.55 (11.26±0.90)
PEDOT:PSS	TACB 20 nm	reverse	20.68 (19.13±0.74)	0.80 (0.78±0.03)	74.95 (76.46±0.96)	12.45 (11.38±0.85)
		forward	20.66 (19.13±0.79)	0.80 (0.77±0.04)	74.49 (75.46±0.51)	12.29 (11.07±0.84)
PEDOT:PSS	TTAI 5 nm	reverse	19.57 (19.42±0.39)	0.86 (0.83±0.03)	74.51 (73.39±0.92)	12.50 (11.84±0.37)
		forward	19.29 (19.17±0.47)	0.85 (0.83±0.03)	73.75 (73.38±0.94)	12.14 (11.62±0.33)
PEDOT:PSS	TTAI 10 nm	reverse	20.89 (21.29±0.32)	0.91 (0.87±0.05)	74.83 (73.47±0.84)	14.22 (13.66±0.78)
		forward	20.67 (21.05±0.30)	0.89 (0.85±0.04)	74.20 (73.69±0.66)	13.69 (13.20±0.61)
PEDOT:PSS	TTAI 15 nm	reverse	21.77 (20.68±0.64)	0.86 (0.85±0.04)	72.23 (72.49±1.19)	13.55 (12.71±0.79)
		forward	21.13 (20.31±0.64)	0.85 (0.84±0.04)	72.90 (72.82±0.86)	13.04 (12.35±0.57)
PEDOT:PSS	TTAI 20 nm	reverse	20.04 (19.32±0.48)	0.91 (0.86±0.05)	76.47 (74.59±1.75)	13.88 (12.48±1.16)
		forward	19.83 (19.17±0.43)	0.89 (0.84±0.04)	75.32 (73.59±1.44)	13.31 (11.91±0.97)

Table S4. Photovoltaic parameters at different HIM thicknesses for PTAA/HIM bilayers.

HTL	HIM	Scan direction	J_{SC} [mA/cm^2]	V_{OC} [V]	FF [%]	PCE [%]
PTAA		reverse	22.55 (21.40±0.67)	1.04 (1.02±0.01)	65.33 (65.46±2.18)	15.31 (14.34±0.78)
		forward	23.32 (22.97±0.94)	1.05 (1.04±0.01)	68.18 (67.02±1.40)	16.67 (15.96±0.69)
PTAA	TACB 10 nm	reverse	21.01 (21.37±0.88)	1.06 (1.03±0.02)	68.98 (67.21±1.81)	15.42 (14.80±0.39)
		forward	22.55 (22.09±0.88)	1.06 (1.03±0.04)	69.99 (68.36±2.01)	16.01 (15.23±0.77)
PTAA	TACB 15 nm	reverse	20.66 (19.87±0.54)	1.07 (1.04±0.02)	71.35 (68.95±3.97)	15.85 (14.23±1.13)
		forward	21.52 (21.06±0.82)	1.07 (1.05±0.01)	71.18 (69.55±3.60)	16.36 (15.31±0.72)
PTAA	TACB 20 nm	reverse	22.82 (21.96±1.04)	1.01 (1.03±0.09)	61.86 (63.84±2.15)	14.30 (12.71±0.61)
		forward	23.24 (22.00±1.16)	1.03 (1.03±0.06)	65.48 (66.01±1.73)	15.68 (15.00±0.65)
PTAA	TTAI 5 nm	reverse	22.90 (22.29±0.56)	1.08 (1.02±0.05)	66.07 (65.16±6.32)	16.29 (14.78±1.35)
		forward	22.90 (22.33±0.60)	1.07 (1.00±0.98)	67.42 (64.05±3.41)	16.61 (14.28±1.89)
PTAA	TTAI 10 nm	reverse	22.21 (21.24±1.36)	1.07 (1.00±0.03)	70.28 (66.68±4.84)	16.66 (14.87±2.16)
		forward	22.44 (22.05±0.68)	1.07 (1.05±0.03)	72.95 (68.33±6.42)	17.44 (15.82±2.15)
PTAA	TTAI 15 nm	reverse	23.18 (21.49±0.87)	1.04 (1.03±0.11)	63.30 (65.42±1.58)	15.26 (14.41±0.62)
		forward	24.27 (22.43±1.12)	1.05 (1.04±0.07)	66.05 (67.36±1.44)	16.86 (15.69±0.69)
PTAA	TTAI 20 nm	reverse	22.03 (20.82±0.82)	1.04 (1.02±0.17)	65.65 (65.53±3.36)	15.06 (13.74±1.04)
		forward	22.63 (22.41±0.83)	1.05 (1.04±0.08)	68.49 (66.62±3.03)	16.27 (15.45±0.48)

10.-Devices electroluminescence

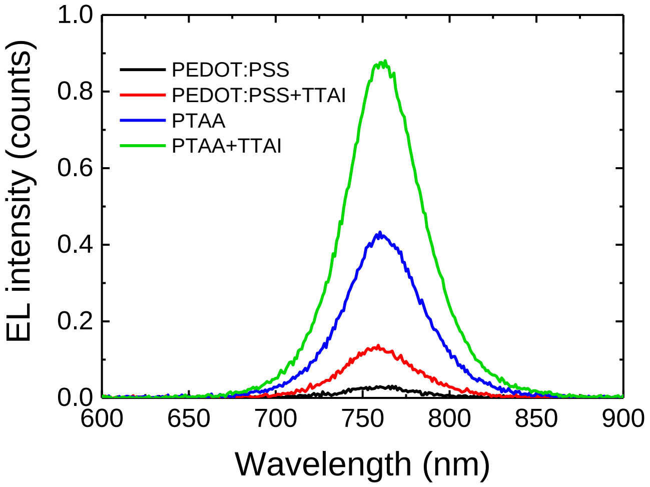

Figure S15. Electroluminescence spectra registered at an applied constant forward voltage of 1.3 V.

11.-References

[1] S.-M. Yoo, S. J. Yoon, J. A. Anta, H. J. Lee, P. P. Boix, I. Mora-Seró, *Joule* **2019**, *3*, 2535-2549.

[2] a) F. Neese, *WIREs Comput. Mol. Sci.* **2012**, *2*, 73-78; b) F. Neese, *WIREs Comput. Mol. Sci.* **2018**, *8*, e1327.

[3] a) C. Lee, W. Yang, R. G. Parr, *Phys. Rev. B* **1988**, *37*, 785-789; b) A. D. Becke, *J. Chem. Phys.* **1993**, *98*, 5648-5652.

[4] F. Weigend, R. Ahlrichs, *Phys. Chem. Chem. Phys.* **2005**, *7*, 3297-3305.

[5] F. Neese, F. Wennmohs, A. Hansen, U. Becker, *Chem. Phys.* **2009**, *356*, 98-109.

[6] E. Caldeweyher, C. Bannwarth, S. Grimme, *J. Chem. Phys.* **2017**, *147*, 034112.

[7] V. Barone, M. Cossi, *J. Phys. Chem. A* **1998**, *102*, 1995-2001.

[8] a) B. J. Bruijnaers, E. Schiepers, C. H. L. Weijtens, S. C. J. Meskers, M. M. Wienk, R. A. J. Janssen, *J. Mater. Chem. A* **2018**, *6*, 6882-6890; b) H. Yoshida, *J. Phys. Chem. C* **2015**, *119*, 24459-24464; c) H. Yoshida, *J. Phys. Chem. C* **2014**, *118*, 24377-24382.
